# Spray Coating Oxidized Graphene-Based Materials Makes Polyurethane Surfaces Antibacterial and Biocompatible: A Promising Approach for Medical Devices

**DOI:** 10.1021/acsami.4c20015

**Published:** 2026-01-20

**Authors:** Natacha Rosa, Inês Borges, Patrícia C. Henriques, Andreia T. Pereira, Rita N. Gomes, Fernão D. Magalhães, Inês C. Gonçalves

**Affiliations:** † LEPABE − Faculdade de Engenharia, 26706 Universidade do Porto, Rua Dr. Roberto Frias, 4200-465 Porto, Portugal; ‡ i3S − Instituto de Investigação e Inovação em Saúde, 26706 Universidade do Porto, Rua Alfredo Allen, 208, 4200-135 Porto, Portugal; § INEB − Instituto de Engenharia Biomédica, 26706 Universidade do Porto, Rua Alfredo Allen, 208, 4200-135 Porto, Portugal; ∥ ALiCE − Associate Laboratory in Chemical Engineering, Faculty of Engineering, University of Porto, Rua Dr. Roberto Frias, 4200-465 Porto, Portugal

**Keywords:** oxidized graphene nanoplatelets, graphene oxide, polyurethane, bacteria, spray coating

## Abstract

Preventing bacterial infections remains a primary concern for healthcare systems and patients. Polyurethane (PU) is widely used in various invasive medical devices, improving PU surfaces—particularly by reducing bacterial adhesion—a highly sought-after goal. This study explores the spray coating of PU-based medical devices with oxidized graphene-based materials (GBMox) as antibacterial agents, specifically oxidized graphene nanoplatelets (GNPox) and graphene oxide (GO). Different formulations, with and without PU as a coating binder, were investigated to assess the potential for tuning the coating based on the intended application. The resulting coatings were characterized in terms of morphology using Atomic Force Microscopy (AFM) and Scanning Electron Microscopy (SEM), along with wettability and stability analysis. Antibacterial activity was evaluated against *Staphylococcus epidermidis*, with GNPox-containing coatings reducing bacterial adhesion by up to 82%, with no effect from blood plasma proteins. *In vitro* biocompatibility was confirmed across all surfaces: human fibroblasts adhered and spread on coatings without PU, while coatings combining GNPox and PU retained the low cell adhesiveness typical of PU. Overall, GBMox coatings on PU enhance antibacterial performance, preserve biocompatibility, and enable tuning of the cell adhesion behavior. These coatings offer a promising antibiotic-free strategy for PU-based biomedical applications, and the spray coating method provides a simple, reproducible, and industrially scalable approach.

## Graphical abstract

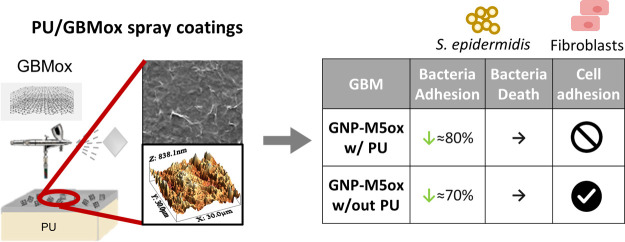

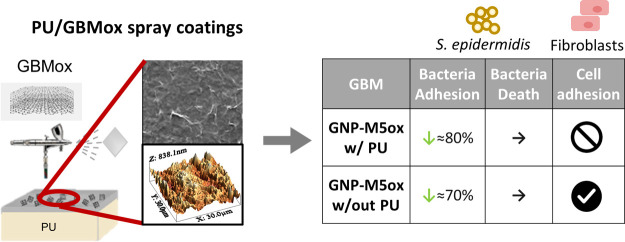

## 1 Introduction

In current days, more attention and concern are paid to the treatment and prevention of bacterial infections since they have presented a significant challenge and burden to the healthcare system.^1^ Antibiotics, the most effective treatment for bacterial infection, have raised concerns in clinical practice because of the side effects and the emergence of highly resistant strains of microorganisms.^1^ Hence, it is critical to advance new strategies to prevent bacterial infections and replace antibiotic treatments. In the prevention setting, antimicrobial materials have a leading role, with a registered increase in the demand and research in the polymers field.^2^ The latter market integrates several sectors, such as medical and healthcare, personal care, packaging, food processing, infant care, building and construction, and automotive, among many others.^3^ However, healthcare, including medical devices and biomaterials, represents the most significant share.^2^

Polyurethane (PU) is among the most widely used polymers for preparing implants and medical devices, despite being very susceptible to microbial attack.^4,5^ This polymer is widely employed in blood-contacting medical devices, such as implanted and interventional catheters, due to its exceptional mechanical strength. This property enables the fabrication of catheters with thin walls that maintain longitudinal rigidity and resist lumen collapse under high negative pressures. Additionally, the material exhibits relatively good hemocompatibility and tissue compatibility, further supporting its use in intravascular applications.^6^ Catheters are essential for chemotherapy, antibiotics, blood, and nutritional support. Unfortunately, they are also prone to infections that cause high rates of morbidity and mortality and high healthcare costs as a result of treatment, testing, and prolonged duration of hospital stay.^4^ Hence, this makes vascular access a determinant factor in a patient’s healing outcome and the prevention of catheter-associated infections a crucial concern. Bacterial colonization of the catheter which occurs during catheter insertion or from bacteria migration at the catheter–skin interface is the major source of infection.^7^ Hence, the duration of catheterization has a direct influence on the likelihood and type of infection.^8^ Dermal microorganisms, particularly *Staphylococcus* and *Streptococcus* species, are predominantly responsible for the majority of these infections, and treatment is mostly performed by antibiotic therapy or, in more severe cases, catheter removal.^9^

In an attempt to reduce catheter-related infection rates, several antibacterial coating approaches have been developed, being either antiadhesive or bactericidal, namely, heparin-coated catheters,^10^ antimicrobial-coated catheters through the use of antimicrobial metal and metal oxides nanoparticles (e.g., ZnO, Ag, Cu, Fe_3_O_4_, Al_2_O_3_, TiO_3_, and SiO_3_),^7^ and antibiotic-impregnated catheters.^11^ Although these prevention strategies are capable of reducing the incidence of catheter-related infections, they have also demonstrated limitations. Heparin inhibits bacterial adhesion and biofilm formation with up to 90% adhesion reduction rates. Metal nanoparticles, such as silver and copper, disrupt bacterial membranes and generate Reactive Oxygen Species (ROS), achieving an over 90% bacterial reduction. Antibiotics target bacterial processes such as protein synthesis, reducing planktonic bacteria by 80–95%, though resistance and biofilm penetration issues remain concerns. However, heparin leads to bleeding risk due to its anticoagulant nature, while metal and metal oxide nanoparticles pose toxicity and environmental risks and are costly to produce. Despite their strengths, antibiotic-impregnated catheters often fail against biofilms and can cause patient side effects.^1,12,13^ The performance of antibacterial coatings is significantly limited by trade-offs between thin and thick applications and have a risk of associated bacteria resistance, which is a major public health issue.^14^ Thin coatings exhibit limited antibacterial efficacy, whereas thicker coatings, while offering prolonged durability, are more prone to cracking, peeling, and flaking. Besides these, other more recent strategies have been explored to develop antibacterial materials, including surface nanopatterning (such as nanopillars),^15^ self-healing superhydrophobic coatings,^16^ and smart antibacterial surfaces with switchable bacteria-killing and bacteria-releasing capabilities, as well-reviewed by Wei et al.,^17^ and with additional antifouling function as demonstrated by Pan et al.^18^ However, they also have limitations such as scalability, large-scale production costs, and efficiency concerns that hinder clinical transfer, despite promising antimicrobial properties and demonstrated biocompatibility.^19^ Hence, despite these improvements, an optimal strategy for long-term catheter infection prevention has not yet been developed.

Graphene, a novel class of two-dimensional carbon nanostructure, has recently garnered significant attention from the scientific communities due to its unprecedented properties.^20^ Graphene derivatives such as oxidized graphene nanoplatelets (GNPox) and graphene oxide (GO) present good hydrophilicity and biocompatibility due to the oxygen-containing functional groups. Such properties led to the exploration of numerous applications in cutting-edge biological and medical sciences fields. Promising studies on graphene-based materials (GBMs) have been reported of the use of these nanomaterials as antibacterial agents.^21–23^ The biocidal effect of GNPox and GO has been associated with sharp edges of nanosheet-induced membrane stress. This can lead to physical damage to cell membranes and consequently disruption of bacterial membrane integrity and the leakage of RNA.^21^ It has also been suggested that GBMs may induce oxidative stress,^21^ either through ROS-dependent or ROS-independent pathways.^24^ Studies indicate that the antimicrobial properties of graphene materials depend on their physicochemical or structural characteristics such as lateral size, layer count, morphology, and dispersibility.^21,25,26^ By proper control of these factors, superior inhibition of bacteria adherence and growth or induction of bacterial death can be attained. Therefore, this type of relatively low-cost and effective carbon nanomaterial shows promise as an antibacterial agent.

In previous studies, we have produced coatings applied in polymers, namely, dip coating of PU.^27^ The coating formulations contained a polymeric binder in solution to ensure the adhesion of the particles onto the surface. Regarding the work developed by Borges et al.,^27^ PU surfaces dip coated with oxidized GNP showed 70% reduction in bacterial adhesion and bacterial death. However, in terms of practical applications, dip coating has disadvantages, namely, the undesirable material waste.^28^ In this study, we evaluate the possibility of using spray coating to provide antimicrobial properties to PU-based medical devices, namely, for the incorporation of oxidized graphene-based materials (GBMox) as antibacterial agents. Specifically, GNPox and GO are explored using different formulations with and without PU as a coating binder.

Spray coating is under investigation as a promising technique that is still in an emerging stage for this specific application. Its superiority is related to its low material waste, strong reproducibility, high deposition rates, and reduced cost, which result in a throughput procedure, with potential as an industrially scalable process.^28^ Tecothane, a medical grade thermoplastic PU, is preferentially used in invasive devices designed for blood-contacting applications (e.g., vascular catheters, breast implants, heart valves, and vascular grafts), and was selected as reference material, based on its high hydrophilicity and excellent biocompatibility.^7^ Oxidized GBMs were selected due to their higher antibacterial and biocompatibility properties compared to nonoxidized GBMs.^22,29^

In this study, GO and GNPox were employed to investigate how the thickness of GBMs influences their antibacterial activity when they are applied to surfaces.

Dissolved PU was considered as an adhesion promoter, being used as a binding agent to enhance the attachment of the GBMox to the substrate. The possibility of it influencing the antibacterial activity of the GBMox, by depositing on their surface and therefore hindering direct contact with bacteria, was also evaluated. The GBMox particles were applied by airbrush spray coating, to obtain a reproducible, industrially scalable, and simple procedure for covering all types of PU surfaces, like catheters. Hence, the antibacterial activity and biocompatibility of PU films coated with GO and GNPox, with and without PU binder, were evaluated and compared to those of PU substrates without such coatings.

## 2 Materials and Methods

### 2.1 Materials

Graphene nanoplatelets (xGnP Graphene Nanoplatelets grade M) with an average lateral size of 5 mm (GNP) were purchased from XG Sciences (Lansing, MI, USA). Graphite was purchased from the Cabot Corporation (Boston, MA, USA). Dimethylformamide (DMF) and H_3_PO_4_ were purchased from Sigma-Aldrich (St. Louis, MO, USA). Tetrahydrofuran (THF) was purchased from JMGS (Portugal). H_2_SO_4_ (95–97%) was purchased from VWR (Darmstadt, Germany). All reagents were used as received. Tecothane aromatic grade TT-1095A was commercially obtained from Lubrizol Life Sciences (Wickliffe, OH, USA) in the form of colorless pellets. Tecothane is medical grade polyurethane and follows standards for +99% purity and low residual monomers <0.1% without toxic additives in accordance with ISO 10993. The product was handled and treated as received from the manufacturer, without any treatment and according to the supplier’s processing recommendations.

### 2.2 Production of GNPox- and GO-Coated Polyurethane Films

#### 2.2.1 GNPox and GO Production

GNPox was prepared according to the modified Hummer’s method, as reported previously,^22^ considering a 1:6 ratio of GNP:KMnO_4_. Briefly, 3 g of GNP was added to a mixture of 120 mL of H_2_SO_4_ (95–97%) and 30 mL of H_3_PO_4_ at room temperature (RT) and with stirring. The solution was cooled to 0 °C using an ice bath, and then 18 g of KMnO_4_ was gradually added. The mixture was kept at 35 °C and stirred for 2 h. Afterward, and at 0 °C, 450 mL of distilled water (*d*H_2_O) was slowly added while stirring. To terminate the reaction, the resulting solution was treated with 35% H_2_O_2_, until oxygen release ended. The obtained GNPox was washed with dH_2_O and centrifuged at 4000 rpm for 30 min, and the supernatant was decanted. This step was repeated until the washing water had a pH close to that of *d*H_2_O (pH ∼ 5).

GO was also prepared according to the modified Hummer’s method, described above, using commercial graphite as the starting material. The purified suspension after washing was then subjected to ultrasonic bath sonication (ATM40-3LCD, Ovan, Barcelona, Spain) for 6 h to exfoliate oxidized graphene sheets inside the graphite structure, which allows for producing a stable suspension of graphene oxide in water. The ultrasonic bath used for oxidized graphene exfoliation has a power of 100 W. Ultrasound is a well-known approach for promoting the separation of GO sheets present in the oxidized graphite structure and is considered part of the modified Hummer’s method protocol. The stability of the obtained GO aqueous dispersion comes from the presence of oxygen-containing groups, which provide a hydrophilic character to the oxidized graphene surface.

#### 2.2.2 Polyurethane Film Production

The PU films were produced by hot-pressing using a metallic sheet frame with 0.5 mm thickness. The frame was placed on the press’s lower plate and preheated to 210 °C, on top of a Teflon film and a metal sheet, and then 15 g of Tecothane pellets was layered at the center. In order to induce polymer melting, the hot-press upper plate, also at 210 °C, was lowered until getting close to the pellets (without direct contact) and kept in that position for 10 min. Afterward, a second Teflon film and a metal sheet were placed over the melted pellets, the top plate was lowered until contacting the metal frame, which was placed over the melted pellets, and the top plate was lowered until contacting the metal frame. The structure was then pressed for 5 min. The assembly was removed from the hot press and quenched in water for 3 min. The hot-pressed PU film was then removed. All hot-pressed PU films were visually verified for defects, such as bubbles and inconsistencies in texture. The thickness was verified with the use of a caliper. The samples that did not follow the conformity were discarded. Samples of 30 × 30 mm were cut and used as spray coating substrate.

#### 2.2.3 Coating of Polyurethane Films with GNPox and GO

Fifteen milligrams of the produced GNPox or GO was dispersed in 30 mL of THF through ultrasonication (UIP1000hd, Hielscher, Teltow, Germany) for 90 s in an ice bath. The ultrasonic probe used for dispersing GNPox and GO in THF, for coating preparation, has a power of 1000 W. Here, the objective was to effectively deagglomerate the particles and ensure that they would be uniformly individualized in the coating. This dispersion was added to 20 mL of THF (GNPox/THF or GO/THF) or 19.7 mL of THF and 0.3 mL of a mixture of 0.5 mg/mL PU in DMF (GNPox/THF+PU or GO/THF+PU) and ultrasonicated for 90 s in an ice bath. The last step was repeated three times. Since a high-power probe was used, repetition of the probe ultrasonication step avoided overheating the liquid dispersion. Thus, using three 90 s ultrasonication steps, with intermediate cooling, ensured effective dispersion without attaining high temperatures that would cause solvent evaporation and possible changes in the oxidation state of the particles. The freshly ultrasonicated solutions were kept dispersed through magnetic stirring in a closed flask to avoid solvent evaporation. Control PU films coated solely with THF and with PU dissolved in THF (THF+PU) were also prepared.

For spray coating, a 0.2 mm nozzle airbrush (Quadrimovel, Agualva-Cacém, Portugal) was used at a distance of 10 cm from the film’s surface. Nitrogen was used as carrier gas, at a pressure of 1.7 bar. Six milliliters of dispersion was sprayed on the 30 × 30 mm PU films inside a fume hood. The hand-held nozzle was kept perpendicular to the vertical film surface, and the spray was applied with repetitive horizontal angular movements, guaranteeing homogeneous coating application. The coated samples were then dried at room temperature overnight.

### 2.3 GNPox and GO Powder Characterization

#### 2.3.1 X-ray Photoelectron Spectroscopy (XPS)

X-ray photoelectron spectroscopy (XPS) was used to analyze the atomic compositions of GNPox and GO sheets. XPS analysis was performed at CEMUP (Centro de Materiais da Universidade do Porto) using Kratos Axis Ultra HAS (Kratos Analytical, Manchester, UK) equipment. For analysis, an achromatic Al X-ray source operating at 15 kV (90 W) was used. The survey spectrum was obtained at 80 eV and the elemental high-resolution spectra (C, O, and Si) at 40 eV. The analysis of the GNP was performed at random points of the samples, focusing on areas of 300 μm × 700 μm. The deconvolution of C 1s and O 1s high-resolution spectra was performed with the CasaXPS software (2.3.16 version, Casa Software Ltd., Teignmouth, UK). Peak fitting was performed using Shirley-type background subtraction and Gaussian–Lorentzian (70:30) peak shape, with exception of the sp^2^ carbon peak, which was fitted using an asymmetric Lorentzian function with an asymmetry parameter of 0.14.^30^ The identification of peak positions was carried out with reference to previously reported literature data.^31^

### 2.4 Coating Morphological and Chemical Characterization

#### 2.4.1 Scanning Electron Microscopy (SEM)

Surface morphology and GBMs distribution of uncoated and coated films were examined by Scanning Electron Microscopy (SEM), using an FEI Quanta 400FEG ESEM/EDAX Genesis X4M with an acceleration voltage of 5 kV, at CEMUP. For coating thickness analysis, samples were frozen in liquid nitrogen and sectioned to evaluate the transversal cut. Samples were mounted on carbon tape and coated with palladium (Pd) and gold (Au) by sputtering using the SPI Module sputter coater to promote surface conductivity. Uncoated PU films were imaged as controls to establish baseline surface morphology for comparison to coated films.

#### 2.4.2 Atomic Force Microscopy (AFM)

Surface morphology and topography of uncoated and coated films were evaluated using the Atomic Force Microscope (AFM) model PicoPlus 5500 (Keysight Technologies, Santa Rosa, CA, USA). Imaging, which was acquired with Picoview 1.20 software version, was performed at RT in intermittent tapping mode (a noncontact mode that is nondestructive for surfaces), using a silicon cantilever tip with a spring constant (*K*) of 26 N/m and *f*_0_ = 300 kHz (Bruker, Billerica, MA, USA). Each data scan was collected over a 30 × 30 μm area at a scanning frequency of 295 Hz. 2D and 3D morphologies and the surface profile were collected and analyzed using the WSxM 5.0 software. The 2D and 3D topography images and the corresponding surface linear profiles were determined. A set of amplitude, hybrid, and special surface parameters which seem to affect initial microbial colonization^32,33^ were calculated, namely, surface roughness (*R*_a_), mean square surface roughness (*S*_q_), 10-point average roughness (*S*_*z*_), skewness (*S*_sk_), kurtosis (*S*_ku_), summit density (*S*_ds_), texture-aspect ratio (*S*_tr_), texture direction (*S*_td_), RMS value of the surface slope (*S*_dq_), mean summit curvature (*S*_sc_), and surface area ratio (*S*_dr_). The reported roughness values are mean values from three images. Statistics were formulated to each parameter using Kruskal–Wallis uncorrected Dunn’s test for multiple comparisons.

#### 2.4.3 Water Contact Angle

The wettability of the samples was evaluated with a goniometer by the sessile drop technique using water as test liquid. The contact angle was determined at RT and humidity by using a video-assisted contact angle measuring device (DataPhysics OCA15 Plus, Filderstadt, Germany) and mapping software (SCA20 software, Filderstadt, Germany). A drop of Milli-Q water (2 μL) was placed on the surfaces, and images were immediately captured using a high-resolution camera. Within 10 s of the introduction of the droplets, the contact angle formed between the sessile droplets and the surfaces was measured. The contact angle is expressed as the mean and standard deviation of three independent measurements taken at three different sites on each surface.

### 2.5 Coating Stability

Coating adhesion was evaluated through the rubbing test, as described by Gomes et al.^22^ A white eraser made of plasticized poly­(vinyl chloride) was rubbed on the samples with constant pressure (nonquantified) to determine if the coating had adhered to the polymeric film surface. The same operator applied a single stroke to ensure repeatability. Identification of coating removal was performed by evaluating the rubber surface and performing SEM analysis of the surface before and after the rubbing test.

### 2.6 Antibacterial Activity Assays

#### 2.6.1 Bacterial Strain and Growth Conditions

*Staphylococcus epidermidis* (ATCC 35984), which causes catheter-associated infections, was clinically isolated from patients and obtained from the American Type Culture Collection. Bacteria were grown in Trypticase Soy Agar (TSA, Merck, Darmstadt, Germany) plates overnight (16 h) at 37 °C and used immediately or stored at 4 °C for up to 1 week. Two colonies were collected, inoculated into 5 mL of Trypticase Soy Broth (TSB, Merck, Darmstadt, Germany), and cultured overnight (16 h) at 37 °C with stirring at 150 rpm.

#### 2.6.2 Bacterial Activity Assessment

Samples were cut into disks of 14 mm diameter, rinsed 3 times with *d*H_2_O, dried with argon (Ar), and sterilized using ethylene oxide. Materials were allowed to equilibrate inside the laminar flow chamber at room temperature overnight prior to use, in order to allow degassing of residual gases. The antibacterial activity of the surfaces was assessed following an adaptation of the standard “ISO 22196 - Measurement of antibacterial activity on plastics and other non-porous surfaces”.^22,27^ The overnight culture of *S. epidermidis* (16 h) was adjusted to a target concentration of 6 × 10^5^ CFUs/mL in fresh TSB medium or fresh TSB supplemented with 10% human plasma, to mimic interaction with blood proteins in catheter surfaces. Bacterial concentration was calculated by OD600 measurement and confirmed by CFU counting of the initial inoculum. A drop of 15 μL of bacterial suspension was placed on top of the surfaces, and a 9 mm diameter polypropylene (PP) sterile film was used to promote contact between the bacteria and the sample surface. Empty wells were filled with sterile *d*H_2_O, and upon incubation, the plates were placed inside a container with moistened paper to avoid evaporation. Samples were incubated for 24 h under static conditions at 37 °C. After incubation, the samples were rinsed with 1.5 mL of fresh TSB to release the PP film and loosely adherent bacteria. The planktonic bacteria present in the supernatant and bacteria adherent to the samples’ surfaces were analyzed separately. Experiments were performed with *n* = 5.

#### 2.6.3 Adherent Bacteria

Bacteria adherent to the surfaces were assessed by staining total and dead bacteria through DNA staining. Samples were rinsed twice with sterile phosphate-buffered saline (PBS) and incubated with 5 μM Draq5 (BioStatus, Shepshed, UK) at RT for 15 min and protected from light for total bacteria staining. Samples were then incubated with 1.25 μg/mL propidium iodide (PI) (Molecular Probes, Waltham, MA, USA) at RT and protected from light for dead bacteria staining. Afterward, samples were rinsed with PBS and transferred to a glass-bottom black 24-well μ-plate (ibidi, Gräfelfing, Germany) with the surface of interest facing the bottom. Images of 9 fields per sample, with 11 to 23 stacks (z-step of 1.5 μm), were acquired using the high-content screening microscope IN Cell Analyzer 2000 (GE Healthcare Life Sciences, UK) in the Cy5 (705 nm) and Cy3 (605 nm) channels with a Nikon 40×/0.95 NA Plan Fluor objective and through 3D deconvolution. Images were then collapsed using IN Cell Developer software, and quantification of bacteria was performed by using Fiji software.

#### 2.6.4 Planktonic Bacteria

Viable cell count was performed using the agar plate culture method. For that, serial dilutions (10^–2^, 10^–4^, 10^–6^) of the supernatants were prepared, and 3 drops of 10 μL of each dilution were plated in TSA. Plates were incubated overnight at 37 °C and CFUs counted. Resazurin was added to the supernatants (10% (v/v)) and incubated for 1 h at 37 °C. The relative fluorescence units (RFUs) were then measured (λex: 530 nm; λem: 590 nm) in a microplate fluorometer (Spectra Max GeminiXS, Molecular Devices, San Jose, CA, USA). The RFUs were correlated to the metabolic activity of the bacteria present in the medium.

### 2.7 Clotting Time Evaluation

Samples were cut into disks of 9 mm diameter, rinsed 3 times with *d*H_2_O, and dried before use. Clotting time was evaluated as in Gonçalves et al.^34^ Samples were placed in 48-well non-tissue culture treated polystyrene (PS) plates, and glass disks (10 mm diameter) and PS (well plate only) were used as positive and negative controls, respectively. Recalcified plasma was prepared by adding a 1 M stock solution of CaCl_2_ to human plasma (citrate-anticoagulated; thawed at 37 °C) to obtain a final calcium concentration of 20 mM. The recalcified plasma was then quickly mixed in a vortex, and 200 μL was immediately added to each well of the 48-well plates. Plates were placed in an incubator at 37 °C with shaking (150 rpm), and the plasma clotting time was visually determined as the time it took for the plasma to undergo gelation (i.e., full coagulation), detected by loss of movement of the plasma in response to shaking.

### 2.8 *In Vitro* Biocompatibility

#### 2.8.1 Cell Lines and Growth Conditions

*In vitro* biocompatibility assays were performed using human fibroblasts HFF1 (ATCC SCRC-1041), grown in Dulbecco’s modified Eagle’s medium (DMEM, Gibco, Fisher Scientific, Leicestershire, UK) supplemented with 10% (v/v) fetal bovine serum (FBS, Gibco, Fisher Scientific, Leicestershire, UK) and 1% (v/v) penicillin/streptomycin (Biowest, Nuaillé, France) (DMEM+), at 37 °C under a 5% CO_2_ humidified atmosphere. The medium was changed twice a week. Cells were detached when 90% confluence was reached using a 0.25% (w/v) trypsin–EDTA solution and resuspended in DMEM+ at cellular density according to the assay. Cells were used until passage 16.

#### 2.8.2 Indirect Contact Assay (Extracts)

The biocompatibility of GBMs-containing spray coatings was evaluated by the incubation of fibroblasts with liquid extracts of materials. For that, material extracts were prepared according to ISO 10993-12 - Biological evaluation of medical devices - sample preparation. Briefly, samples were cut into 5 mm diameter disks, sterilized using ethylene oxide, and incubated with DMEM supplemented with 10% FBS (DMEM+), at 37 °C for 24 h in an orbital shaker with agitation (100 rpm). Extracts of DMEM+ alone and of tissue culture polyethylene terephthalate coverslips (TCPET, SARSTEDT AG & Co, Nümbrecht, Germany) were used as the positive control, while a solution of DMEM supplemented with 10% FBS with Triton X-100 0.1 wt % was used as the negative control of cell viability. Fibroblasts were seeded at a density of 1 × 10^5^ cells/mL in 96-well plates and incubated in DMEM+ at 37 °C under a 5% CO_2_ humidified atmosphere. After 24 h the medium was replaced by material extracts, according to ISO 10993-5- Biological evaluation of medical devices - in vitro cytotoxicity. After 24 h of incubation under the same conditions, cell metabolic activity was evaluated by the resazurin assay. For that, DMEM+ containing 20% (v/v) resazurin was incubated with cells at 37 °C for 4 h, and then RFUs were measured (λex: 530 nm; λem: 590 nm) in a microplate fluorometer (Spectra Max GeminiXS, Molecular Devices, San Jose, CA, USA). Assays were performed with *n* = 5.

#### 2.8.3 Direct Contact Assay

Biocompatibility of the GBMs-containing spray coatings was also evaluated by the incubation of fibroblasts directly on the surfaces. Sterilized material disks of 5 mm diameter were placed in 96-well plates. A drop of 10 μL fibroblast suspension containing 1 × 10^6^ cells/mL (1 × 10^4^ cells/well) was seeded on samples’ surfaces and incubated at 37 °C for 2 h. Afterward, 90 μL of DMEM+ was added to each well, reaching a final cell density of 1 × 10^5^ cells/mL, and then the culture was maintained for 24 h at 37 °C under a 5% CO_2_ humidified atmosphere. After that, cell metabolic activity was evaluated by a resazurin assay, and cell morphology was evaluated by immunocytochemistry. For that, samples were incubated with DMEM+ containing 20% (v/v) resazurin at 37 °C for 4 h, and then RFUs were measured (λex: 530 nm; λem: 590 nm) in a microplate fluorometer (Spectra Max GeminiXS, Molecular Devices). Cells were then rinsed with PBS, and fixation was performed with 4 wt % (v/v) paraformaldehyde (PFA, Merck, Darmstadt, Germany) in PBS for 20 min at RT. PFA was removed, and cells were rinsed with PBS and stored at 4 °C. Cell membrane was permeabilized with Triton X-100 0.1 wt % at 4 °C for 5 min, rinsed with PBS, and incubated with 1:100 dilution of phalloidin (Alexa Fluor 488, Molecular Probes, Waltham, MA, USA) in PBS, for 30 min in the dark at RT, to stain cell cytoskeletal filamentous actin (F-actin). After being rinsed with PBS, cells were incubated with 3 μg/mL of a 4′,6-diamidino-2-phenylindole dihydrochloride (DAPI, Merck, Darmstadt, Germany) solution in PBS, for 15 min in the dark at RT, to stain the cell nucleus. Finally, cells were rinsed and stored at 4 °C in PBS to avoid drying. Images of the surfaces were acquired using a Leica TCS SP5 inverted confocal microscope (Leica Microsystems, Germany) working in the AOBS mode, with the HC PLAN APO CS 10×/0.40 objective and using the Diode 405 nm and Ar 488 nm lasers for DAPI and phalloidin stainings, respectively. Cells were cultured in PS (well plate only) with DMEM+ as positive control and in PS with DMEM+ with Triton X-100 0.1 wt % as a negative control of cell viability. Assays were performed with *n* = 5.

### 2.9 Statistical Analysis Methods

AFM results (*n* = 3) are presented as average and standard deviation. Normality tests were performed using Shapiro–Wilk tests for all the assays. The normality of the population distribution was analyzed, and parametric or nonparametric tests were used for normal or non-normal distributions, respectively. For the clotting time (*n* = 3) and metabolic activity (*n* = 5) studies, where the population was not normally distributed, the Kruskal–Wallis test was employed. For optical contact angle (*n* = 3), where a normal distribution was verified, one-way ANOVA followed by Tukey’s multiple comparison was used. The antibacterial activity measurements (*n* = 5) were analyzed using either the Kruskal–Wallis test or one-way ANOVA followed by Tukey’s multiple comparisons, depending on the normality distribution. Statistical analysis was performed using the software GraphPad Prism 9 for Macintosh. Statistical significance was accepted for *p* < 0.05.

## 3 Results and Discussion

### 3.1 Characterization of GNPox and GO Powders

Tables 1 and 2 show the XPS analysis with GNPox and GO surface chemistry information. The elemental composition of the GNPox and GO, namely, the carbon (C) and oxygen (O) atomic percentages, is represented in Table 1. The oxygen percentage in both materials (around 30%) is in accordance with what is expected for materials oxidized by the modified Hummer’s method.^22^ Table 2 demonstrates the existence of hydroxyl (−OH), epoxy (C–O–C), carbonyl (C=O), and carboxyl (O–C=O) groups in GNPox and GO. GNPox consists of oxidized graphene nanoplatelets (2–10 graphene layers) and GO of oxidized graphene sheets (monolayer). Epoxide and hydroxyl groups are located mainly at their basal planes, and carbonyl and carboxyl groups are located along the edges.^27^ From Table 2 it is also possible to identify that, for both GBMs, epoxy is the most abundant oxygen-containing functional group. According to Li et al.,^20^ the functional epoxy groups (which tend to organize in line and pairs on the basal surface of graphene) are responsible for breaking the C–C bonds of the molecular structure and may also lead to the rupture of the graphene sheets, since they are considered a critical intermediate species in the formation of carbonyl pairs. However, the low atomic percentage of carbonyl groups in both the GNPox and GO microparticles (6.58 and 8.68, respectively) allows one to infer that both GBMs present reduced defects in their structure.

**Table 1:** Atomic Percentage of GNPox and GO Powders Obtained by XPS

	Atomic % concentration	
Element	GNPox	GO
C	67.8	65.0
O	30.5	32.9

**Table 2:** Atomic Percentage of Each Chemical Group of GNPox and GO Attained by XPS High-Resolution Deconvolution

		Atomic % concentration	
Chemical group	Binding energy (eV)	GNPox	GO
C=C (sp2)	284.5	27.81	25.28
C–C (sp3)	285.4	24.36	15.27
C–OH	286.4	1.62	6.72
C–OC	287.1	33.91	43.19
C=O	288.0	6.58	8.68
O–C=O	289.2	6.72	0.85

### 3.2 Physicochemical Properties of the GBMs-Containing Coatings

#### 3.2.1 Surface Topography

Factors such as bacterium and substratum physicochemical properties and environmental conditions dictate the potential for adhesion between a bacterial cell and a substratum.^33^ Figure 1 depicts SEM images of the uncoated and coated PU surfaces.

**Figure 1:**
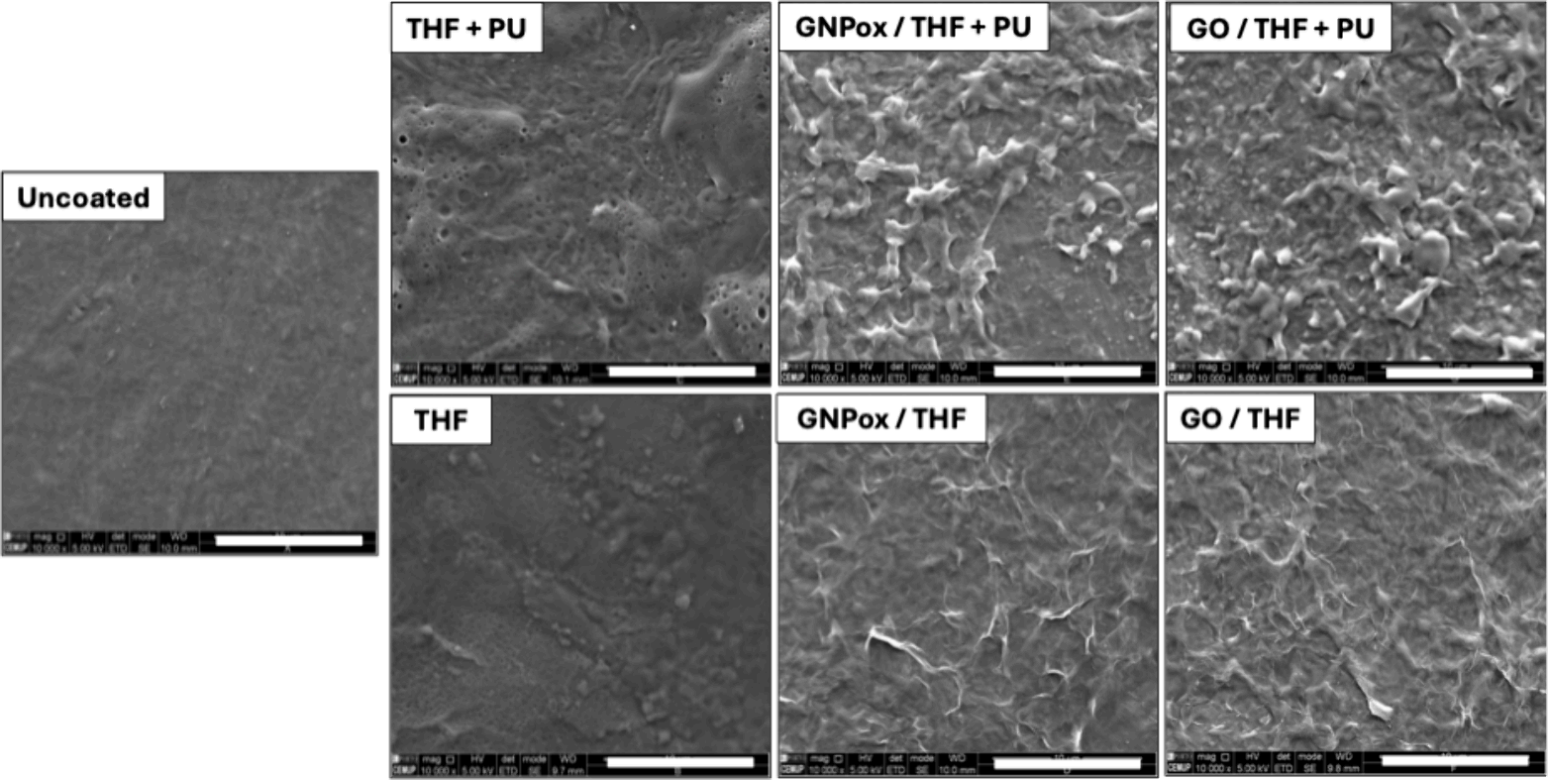
SEM images of uncoated film and THF and THF+PU, GNPox/THF, GNPox/THF+PU, GO/THF, and GO/THF+PU coated films. Images were acquired using secondary electron imaging mode, an Everhart-Thornley detector (ETD), high voltage (HV) of 5.00 kV, 10 000× magnification (mag), and working distance (WD) of ca.10 mm. Scale bar: 10 μm.

Applying a spray containing only with THF slightly altered the surface, resulting from polymer swelling when in contact with THF, a good solvent for PU. When THF+PU was applied, a slightly more irregular surface was obtained, with the dissolved PU depositing on the film surface.

The coatings containing GBMs, GNPox/THF, and GO/THF show a wrinkled surface. Oxidized surfaces, such as GO and GNPox, exhibit a compact and continuous structure, where oxygen-containing groups appear to interact through strong hydrogen bonds. This interaction causes adjacent sheets to “fuse” together, resulting in randomly distributed wrinkles and ridge-like protrusions.^21^ PU inclusion in the coatings agglomerates platelets and decreases the edges. A similar morphology was obtained by other researchers using oxidized GBMs in coatings^22,35,36^ or as free-standing films.^24^ According to Bandeira et al.,^37^ this distorted graphene sheet layout results from attractive interactions between polar groups on the basal surface of the graphene sheets, formed in the oxidation process. When PU was dissolved in the coatings, a more granular surface morphology was visible, in agreement with that of the THF+PU films depicted above. From Figure 1 it is also possible to confirm a homogeneous distribution of the GBMs throughout the substrate. As can be observed in Supporting Information Figure S1, the coating thickness is below μm in size, ranging from 50 to 200 nm.

Bacterial adhesion and colonization are complex processes that depend on many factors, from which surface roughness and hydrophobicity play a determining role.^38^ For the surface topography evaluation, the films were compared by AFM investigations (Figure 2).

**Figure 2:**
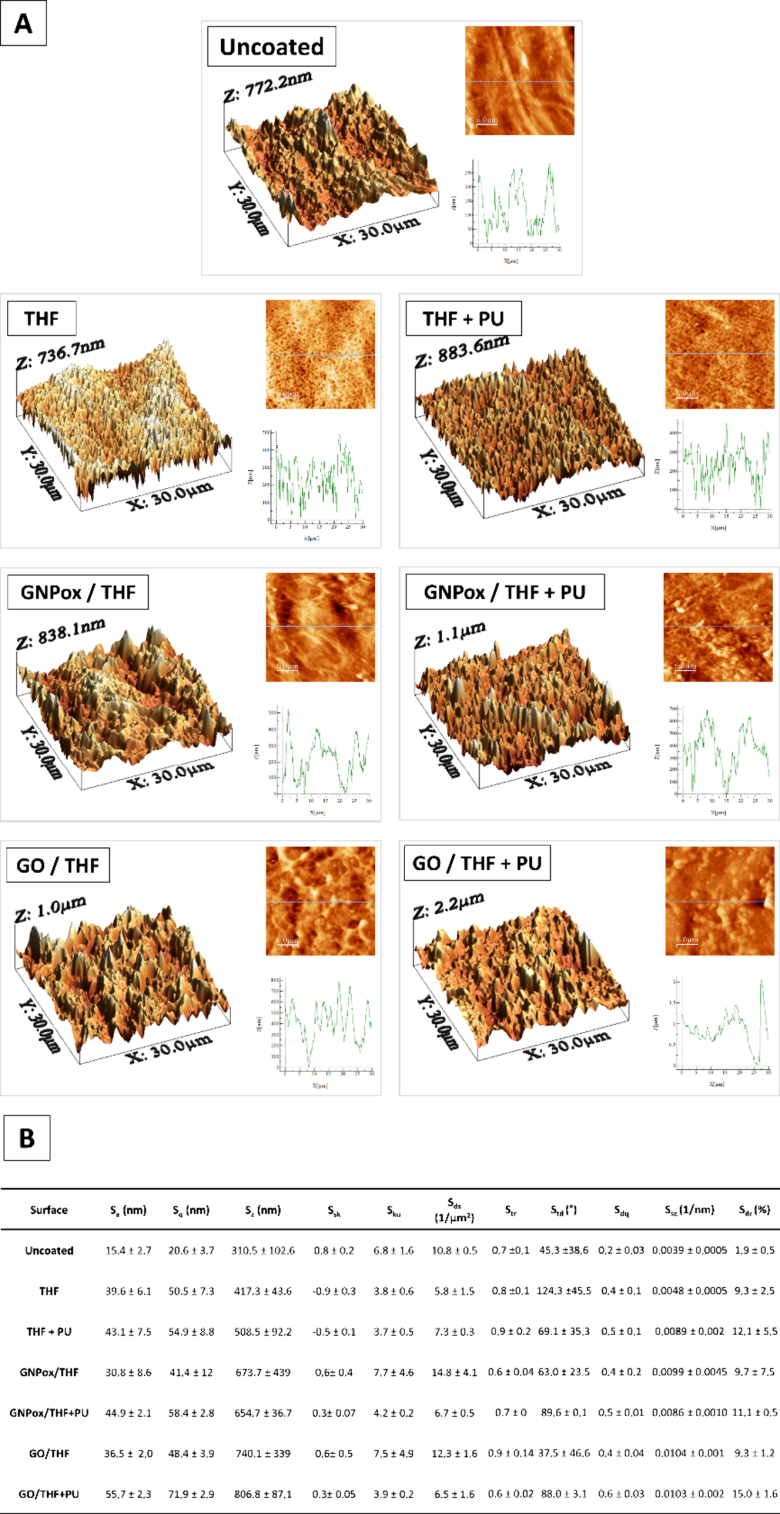
Representative 3D and 2D AFM images and corresponding surface linear profile of films (A, scale bar = 6 μm) and surface amplitudinal and spatial roughness parameters (B) of uncoated and THF, THF+PU, GNPox/THF, GNPox/THF+PU, GO/THF, and GO/THF+PU coated films. The surface roughness parameters considered are the surface roughness (*S*_a_), mean square surface roughness (*S*_q_), 10-point average roughness (*S*_*z*_), skewness (*S*_sk_), kurtosis (*S*_ku_), summit density (*S*_ds_), texture-aspect ratio (*S*_tr_), texture direction (*S*_td_), RMS value of the surface slope (*S*_dq_), mean summit curvature (*S*_sc_), and surface area ratio (*S*_dr_). Data are plotted as mean ± SD. Statistical analysis is presented in Supporting Information table.

Figure 2.B demonstrates that all of the coatings applied led to an increase in specific amplitude surface roughness parameters, such as *S*_a_, *S*_q_, and *S*_*z*_. Surface roughness (*S*_a_) is the most universal parameter for surface topography evaluation, and it characterizes the average deviation of the height values from the mean plane.^33^ The uncoated films present the smoother of all the evaluated surfaces (≈ 15 nm), despite not statistically significant from THF (*p* = 0.07), GNPox/THF (*p* = 0.32), and GO/THF (*p* = 0.18). The coatings with dissolved PU led to an increase of the surface *S*_a_ values, i.e., led to an increase of the surface roughness when compared to the ones where the dissolved PU was not added, despite being only statistically significant (*p* = 0.02) in GO/THF (36.5 nm) vs GO/THF+PU (55.7 nm). Based on the data from Figure 2.B, there is no statistical difference in the *S*_a_ value when GNPox and GO were used, namely, GNPox/THF vs GO/THF (*p* = 0.74) and GNPox/THF+PU vs GO/THF+PU (*p* = 0.32). It is widely accepted that rough surfaces with greater contact area and protective surface defects from shear forces and abrasion (e.g., pits, bumps, or troughs) can influence bacteria adherence at micro- and nanoscale levels.^39^ This was demonstrated by Nguyen et al.,^40^ who showed that nanoscale features (*S*_a_ = 41.1 ± 1 nm) significantly reduce bacterial *P. aeruginosa* (bacteria dimensions: 0.5–1 μm × 1–5 μm^41^) attachment to 57% and *S. aureus* (bacteria dimensions: diameter of 0.5–1.5 μm^41^) to 20%, likely due to increased air–water interfaces limiting bacterial contact. It should be highlighted that, in all the surfaces, the measured *S*_a_ values were under 200 nm, which according to several authors^32,38^ are optimal values for low microbial colonization. Dela-Pinta et al.^42^ evaluated *S. epidermidis* biofilm formation on PU surfaces, verifying that high *S*_a_ values (*S*_a_ = 1880 nm), which are indicators of rougher surfaces, registered the higher biofilm formations. According to Rochford et al.,^43^ surface features of the same magnitude as the adhering bacteria can be a cause for increased bacterial adhesion, where *S. epidermidis* are typically between 500 and 1500 nm in diameter. It has also been stated that rougher surfaces tend to foster microorganisms to accumulate and reside within wide irregular attachment areas such as grooves or pores.^32^ Additional amplitude parameters, namely, the mean square surface roughness (*S*_q_, which represents the root-mean-square deviation from the mean plane within the sampling area^32^) and the 10-point average roughness (*S*_*z*_, which represents the difference between the mean height of the five highest peaks and the mean depth of the five deepest valleys^32^), are in accordance with the height distribution obtained with *S*_a_, with GO/THF+PU having the highest values (*S*_q_ = 71.9 nm and *S*_*z*_ = 806.8 nm), which is significantly different from all samples (*p* < 0.05), except from GNPox/THF+PU. It is important to highlight that the previously considered parameters (*S*_a_, *S*_q_, and *S*_*z*_) give an indication of the height of peaks on a surface; however, they provide no information regarding the peaks’ shape or spatial distribution.^44^

The skewness (*S*_sk_) and kurtosis (*S*_ku_) are amplitude parameters, which allow inferring the spatial variation in height.^32^ Skewness values (*S*_sk_) describe the surface asymmetry from the mean line and where a value equal to 0 represents a Gaussian-like surface.^45^ As shown in Figure 2.B, in almost all of the surfaces evaluated, the surface local maxima dominate over the valleys, which are nicely described by the positive values of the *S*_sk_. In the same way, the negative *S*_sk_ values, which were only registered in the THF and THF+PU coating, indicate a surface-porous sample; that is, the valleys dominate over the peak regimes. The *S*_sk_ value alteration from positive in the uncoated films to negative can be explained by the action of THF at the PU surface. Surface kurtosis (*S*_ku_) gives a measure for the sharpness of the surface height distribution, where a Gaussian value for this parameter is 3.0. All the surfaces evaluated presented *S*_ku_ values higher than 3.0, which refers to surfaces with a spiked high distribution.^45^ The high *S*_ku_ values of GO/THF and GNPox/THF coating, 7.5 ± 4.9 and 7.7 ± 4.6, respectively, show that the coatings include peaks with narrow height distribution.^46^

The number of local maxima (summits) per unit area on a surface is given by the summit density (*S*_ds_), which is the most representative of the hybrid surface features.^32,33^ Besides the number, the form of the summits is also of certain interest. The latter feature can be described by the hybrid parameters mean summit curvature (*S*_sc_) and the RMS value of the surface slope (*S*_dq_).^45^ The mean summit curvature (*S*_sc_) is defined as the average of the principal curvatures of each of the summits on a surface.^33^ From Figure 2.B, it is possible to notice that the surfaces that presented more peaks were the ones coated with GNPox/THF and GO/THF. Also, there is almost a 2-fold increase in the number of peaks in the coatings when only the GBMs were present compared with the samples where the coating had GBMs and dissolved PU. Finally, the applied coating surface summits presented similar curvatures (*S*_sc_) and slopes (*S*_dq_).

The ratio of the surface area to the projected surface area is given by the surface area ratio (*S*_dr_) special parameter. An *S*_dr_ of a completely level surface is 0.^33^ From Figure 2.B it is possible to notice that the *S*_dr_ values increase when dissolved PU is used in the coating, which also follows the tendency for *R*_q_ increase. As stated by Braem et al.,^47^ a higher *S*_dr_ indicates a strong increase in surface area. Another important spatial parameter for assessing the interfacial area between the substratum and microorganisms during initial microbial adhesion is the texture aspect ratio (*S*_tr_ ≤ 1). This parameter characterizes the ratio between the shortest and longest repeating patterns on the surface. An *S*_tr_ lower than 0.3 is an indication that the surface has an oriented and/or periodical structure; that is, the surface texture presents a spatial directionality. An *S*_tr_ value higher than 0.5 indicates the surface isotropy, i.e., has the same characteristics in all directions.^32,33^ Figure 2.B shows that all of the surfaces present an isotropic texture. The high *S*_tr_ values of THF+PU and GO/THF are indicators of a highly isotropic surface. All of the aforementioned spatial parameters describe the substrate contact area provided for microorganisms’ adherence. The texture direction (*S*_td_) determines the angle between the major direction of the surface texture and the *y*-axis of the sampled area. However, this parameter only has a meaning if *S*_tr_ is lower than 0.5 (surface texture spatial directionality), which is not the case for the evaluated surfaces.^33^

#### 3.2.2 Surface Wettability

Surface wettability was investigated by water contact angle, and results are depicted in Figure 3.

**Figure 3:**
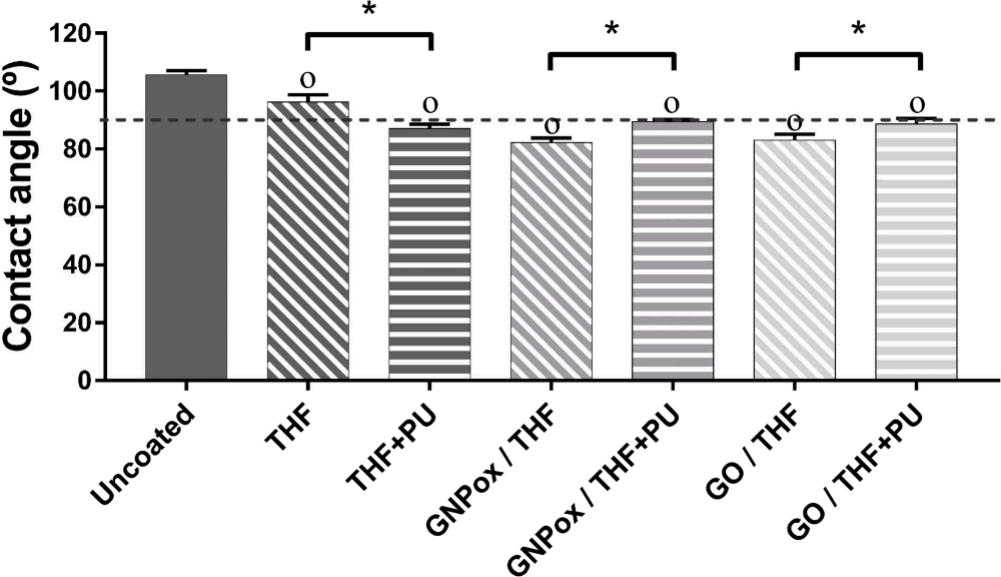
Static contact angle on uncoated and THF, THF+PU, GNPox/THF, GNPox/THF+PU, GO/THF, and GO/THF+PU coated films. The dotted line marks the 90° contact angle (the limit between hydrophobic/hydrophilic surfaces). Symbols indicate statistically significant differences (one-way ANOVA; *p* ≤ 0.05): ^O^ indicates a significant difference from the control PU film (uncoated), and * statistically significant difference between samples.

The contact angle of the uncoated film (105 ± 1.4°) was similar to the values of 102 ± 0.6° obtained previously (despite being obtained by solvent casting).^27^ From Figure 3 it is possible to verify that there is a transition from a hydrophobic surface, for uncoated films, to a hydrophilic surface when the GNPox or GO coating is applied (contact angle of <90°). The hydrophilicity induced by GNPox and GO can be explained by the presence of hydroxyl and epoxide groups on the basal plane and carboxyl groups on the edges.^36^

Figure 3 also shows a statistically significant increase (*p* < 0.05) in the contact angle of the GBMs-coated films when dissolved PU was also applied. This can be explained by the presence of hydrophobic dissolved PU at the surface and/or also by the alteration of surface topography (Figure 2). The latter can be explained by the dependence of wetting on topography. As stated by Ghumatkar et al.,^48^ adding surface roughness decreases the wettability caused by the chemistry of the surface. Following this line of thought, the surfaces will become more hydrophobic when the surface roughness is increased.

The statistically significant difference between the uncoated surface and the THF and THF+PU coated film surface contact angles can only be explained by surface roughness (see Figure 2).

### 3.3 Coating Stability

The strength of a coating adherence was evaluated, by performing a rubbing test, in an attempt to simulate the handling and the insertion of a medical device, such as a catheter, for example.^22^ Morphology variations of the coated surface before and after the rubbing test were evaluated by SEM images, and the results are displayed in Supporting Information Figure S2. The rubbing procedure does not seem to remove the coating, even though a small dragging can be observed in the GNPox/THF+PU coating. It is important to highlight that this test is an aggressive procedure in which rubbing was applied three consecutive times to the coated surfaces, inducing a reasonable amount of shear stress. Hence, the absence of GBMs detachment from the substrate suggests coating stability. The use of dissolved PU in the coating formulation does not seem to improve the GBMs’ adherence to the PU substrate. This can be explained considering that THF, used as the GBMs dispersion medium, swells the PU chains in the substrate, increasing their mobility and promoting their interaction with the nanomaterials. The presence of PU as a binding agent on the dispersion is therefore unnecessary, since the presence of the solvent has maximized the adhesivity of the GBMs on the substrate. After it evaporates, the GBMs are already properly incorporated into the outermost layer of the PU substrate.

Using a solvent compatible with both GBMox and PU enhances coating durability by improving bonding strength between GBMox and PU, reducing risks of delamination and ensuring a uniform structure, minimizing weak points or defects prone to failure, which results in a long-lasting coating for various applications. This durability will also be beneficial for the long-term antibacterial effectiveness of the oxidized GBMs coatings, as GBMox’s structure and properties are stable over time, maintaining its ability to interact with and disrupt microbial membranes.

To the best of our knowledge, this is one of the first studies employing spray coating of GBMs on polyurethanes for antimicrobial purposes. A previous study^49^ used thermal spraying to deposit copper–graphene nanoflakes on stainless steel surfaces; however, no stability tests were conducted. Furthermore, the antibacterial activity assessment was confined to the adherent bacteria. Herein we have also assessed antibacterial activity in suspension bacteria. Additionally, our prior work, Gomes et al.,^22^ demonstrated the effectiveness of spray coating on silicone catheters. Similarly, in this study, spray coating with oxidized GBMs resulted in a stable, rub-resistant coating, making it suitable for coating catheters.

### 3.4 Antibacterial Activity Evaluation

The antibacterial properties of the surfaces toward adherent and planktonic *S. epidermidis* are depicted in Figures 4 and 5, respectively. Information regarding adherent bacteria controls on inert surfaces is present in Supporting Information Figure S3.

**Figure 4:**
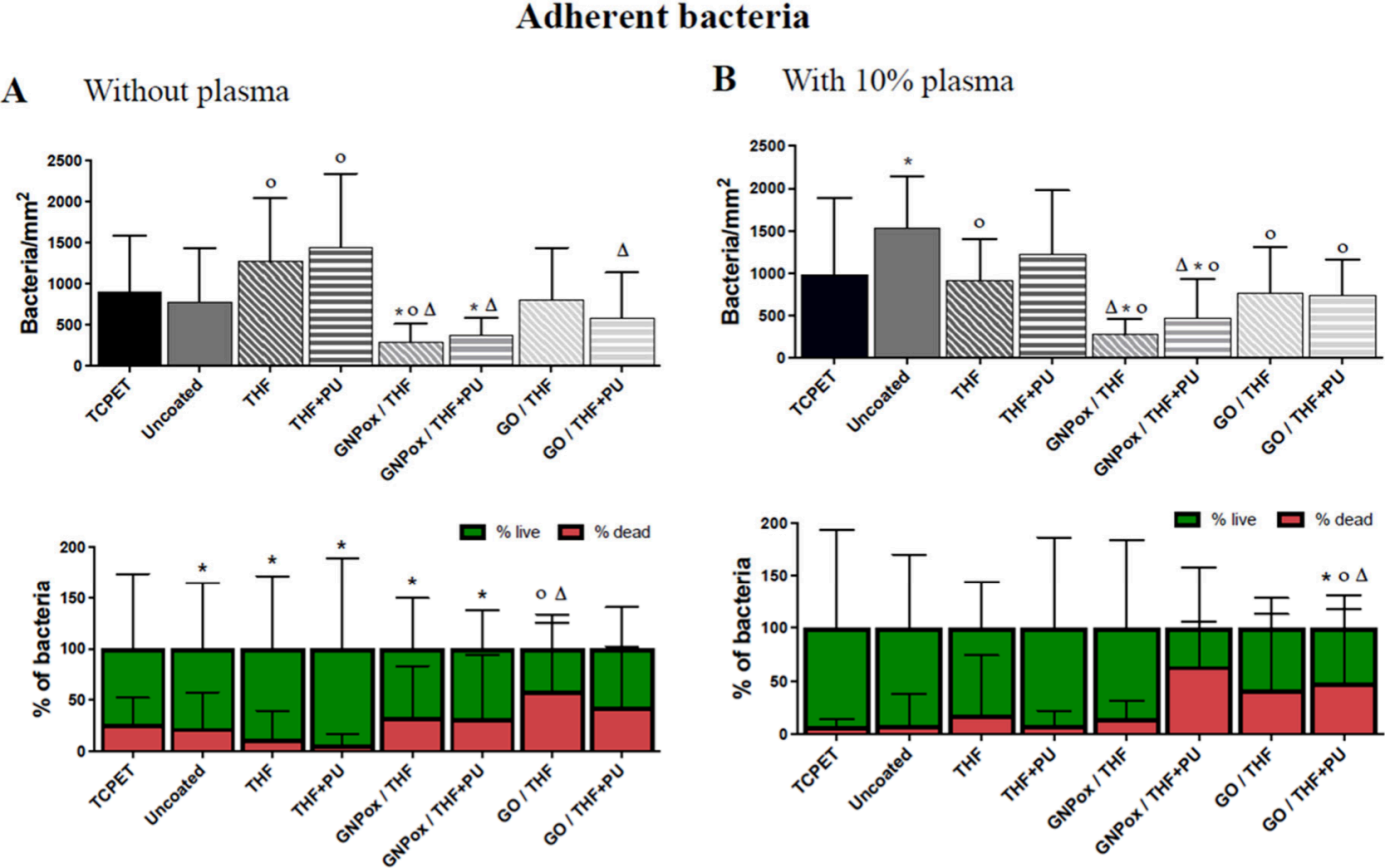
Antibacterial activity of uncoated and THF, THF+PU, GNPox/THF, GNPox/THF+PU, GO/THF and GO/THF+PU coated films toward adherent *S. epidermidis* after 24 h of incubation in unsupplemented culture medium (A) and in medium supplemented with 10% human plasma (B). Symbols indicate statistically significant differences (*p* ≤ 0.05; Kruskal–Wallis test): * indicates a significant difference from TCPET, ^O^ from the control PU film (uncoated), and ^Δ^ from coatings with THF and THF+PU.

**Figure 5:**
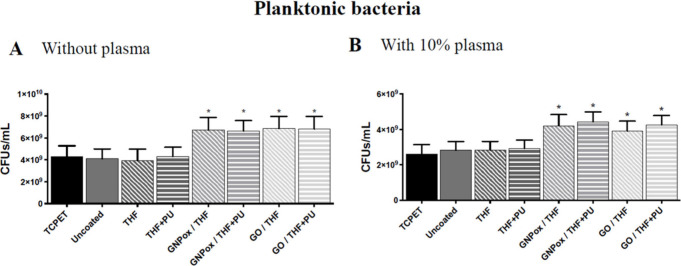
Antibacterial activity of uncoated and THF, THF+PU, GNPox/THF, GNPox/THF+PU, GO/THF, and GO/THF+PU coated films toward planktonic *S. epidermidis* after 24 h of incubation in unsupplemented culture medium (A) and in medium supplemented with 10% human plasma (B). * indicates a statistically significant difference from all control groups (TCPET, uncoated, THF, and THF+PU) (*p* ≤ 0.05; one-way ANOVA (B, D)).

Starting with bacterial adhesion (Figure 4.A), compared to the control PU film (uncoated), a statistically significant decrease (*p* < 0.05) in the number of adhered bacteria was found in the coatings GNPox/THF and GNPox/THF+PU, being 62% and 51%, respectively. In these coatings, the percentage of dead bacteria is maintained. On the other hand, the coatings with GO/THF and GO/THF+PU yielded surfaces with no difference in terms of adhered bacteria compared to PU, but with increased death reaching 59% and 43%, respectively, compared to the percentage in uncoated PU. Antibacterial activity was also evaluated in the presence of plasma proteins, by supplementing the medium with 10% human plasma, and similar results were obtained (Figure 4.B). The coatings GNPox/THF and GNPox/THF+PU decreased bacterial adhesion by around 82% and 69%, respectively, without influencing bacterial death. On the other hand, the coatings GO/THF and GO/THF+PU decreased bacterial adhesion by around 50% (*p* < 0.05), while also increasing the percentage of death to 41% and 48%. Interestingly, surfaces with GBMs-containing coatings presented better antibacterial effects in the presence of human plasma.

Regarding planktonic bacteria (Figure 5), the increased number of CFUs/mL detected in the supernatant indicates an increase (*p* < 0.05) in the number of viable bacteria present in the supernatant (Figure 5.A and B). Besides CFU, the planktonic bacteria were also analyzed in terms of metabolic activity by an Alamar Blue assay, and a small increase was observed in the coatings compared to that of bare PU (Supporting Information Figure S4). Planktonic bacteria are, therefore, viable and metabolically active.

Overall, independently of the presence of PU in the coating solution, the antibacterial effect of coatings with GNPox was reflected in the decrease of bacterial adhesion (antiadhesive), while coatings with GO exerted more effect in bacteria killing while also being antiadhesive. It was also demonstrated that the use of PU in the coating solution, under the conditions tested, did not significantly influence the antibacterial properties of the surfaces. Moreover, in the presence of human plasma and, therefore, in conditions more similar to the human body and to the biomedical applications envisioned for the materials, the antibacterial properties of the GBMox are not masked or compromised. In fact, as highlighted in a previous study from Henriques et al.,^50^ highly oxidized graphene surfaces, such as the ones of herein presented GO and GNPox coatings, tend to reduce the adsorption of human plasma proteins compared to their less oxidized counterparts, including reduced GO (rGO) and few-layer graphene (FLG). This has a direct impact on the overall antibacterial performance of the surfaces—including both antiadhesive properties and bactericidal effects—as plasma proteins often mediate bacterial adhesion through interactions with bacterial adhesins.

*S. epidermidis* was chosen as the model organism in this study due to being the most prevalent pathogen in hospital-acquired infections. Findings from *S. epidermidis* can often be extrapolated to other coagulase-negative *Staphylococcus* species, which are also significant in hospital infections.^51^

Surface topography and roughness modulate bacteria attachment,^33^ and it is also known that the interaction of bacteria with GBMs depends on the orientation of the platelets relative to the bacteria.^21^ Hence, the way GBMs are disposed of on the surfaces and how they are produced influence the antimicrobial properties of the surfaces. In this work, the coatings produced presented irregular or “random” surface topographies. All the surfaces had *S*_a_ values under 200 nm, which are indicative of low microbial colonization. Surfaces with topographic features at the micrometric scale favor attachment since microbial cells tend to maximize contact area with the surface. On the contrary, surfaces with topographic features of dimensions in the submicrometric or nanometric range (much smaller than microbial cells) have been reported to inhibit attachment by reducing the contact area between bacterial cells and the surface.^52,53^ Accordingly, Feng et al. impaired the attachment of *S. epidermidis*, among other bacteria, by producing alumina surfaces with pore sizes of 15 and 25 nm in diameter compared to surfaces with larger pores.^52^

The most significant differences in *S*_a_, *S*_q_, and *S*_*z*_ values are obtained between PU vs GO/THF+PU (*p* = 0.0004 for *S*_a_) and PU vs GNPox/THF+PU (*p* = 0.01 for *S*_a_), which supports that an increase in roughness prevents bacterial adhesion. Although, this increase in roughness seems to correlate with bacterial adhesion prevention, these surfaces are still in the nanometric range. *S*_sc_ was the parameter in which the better antibacterial surface (GO/THF) with a killing effect differed most from that of PU. A smaller *S*_sc_ value indicates surfaces with rounded shapes, and a larger value indicates pointed shapes. In this case, GO/THF has pointed shapes (*S*_sc_ = 0.0104 nm^–1^) compared to PU (*S*_sc_ = 0.0039 nm^–1^) (*p* = 0.0038), which supports bacterial death by sharper edges of GO. Additionally, the wrinkled surface obtained with the use of GNPox and GO compared to the uncoated PU substrate can also facilitate the antibacterial action of the platelets. Zou et al.^54^ studied the influence of the wrinkled surface geometry of GO films on the bacterial viability of *E. coli*, *Mycobacterium smegmatis*, and *S. aureus*. They state that the antibacterial mechanisms of the films result from the formation of a mechanically robust GO surface “trap” that incites the interaction of bacteria with the diameter-matched GO sink, which, afterward, leads to bacterial cell membrane damage.

Hydrophobic force determined by surface physicochemical properties also plays a key role in microorganisms’ adhesion. Bacteria are more likely to adhere to hydrophobic than to hydrophilic surfaces,^32^ and cell surface hydrophobicity tends to increase adhesion. Parra et al.^55^ have shown that the increased hydrophobicity of graphene coatings, as-grown FLG, and transferred graphene onto Ni foils increased the adhesion and viability of *E. coli* cells.^55^ In contrast, using oxidized GBMs increases hydrophilicity and hence should have the opposite effect.

In this work, the wettability of the coatings changed when GNPox and GO were used, with and without dissolved PU. These coatings were shown to be hydrophilic by presenting a water contact angle below 90°, in opposition to the known hydrophobicity of PU (105°) (*p* < 0.05). As such, this increase in hydrophilicity prevented *S. epidermidis* from adhering to surfaces.

Finally, the coatings’ antibacterial effects observed toward *S. epidermidis* can be attributed to the oxidized GBM’s inherent antibacterial activity. An increasing number of studies describe the antibacterial activity of surfaces containing GO, reducing bacterial growth or killing, attributed mainly to the induction of oxidative stress.^26^ Perreault and co-workers investigated the size-dependency of GO antimicrobial activity in suspension and surface coatings by ranging the GO sheet area from 0.01 to 0.65 μm^2^. In GO-coated surfaces, formed by vacuum filtration, they found a higher antimicrobial effect toward *E. coli* with smaller GO sheets through disruption of cell integrity, attributed to oxidative mechanisms associated with the higher defect density of smaller sheets.^26^ The same effects were reported by Shih and co-workers when studying the addition of GO and rGO to bioactive glass and observing much higher antibacterial activity toward *E. coli* using GO (≈ 83%).^56^ Their study attributes the GO antibacterial effects to its higher degree of structural disorder that provides more sites (defects or oxygen vacancies) for ROS production, which would lead to more bacteria death.

Accordingly, upon comparison of spray coatings with GNPox and GO, they both decreased *S. epidermidis* bacterial adhesion, with GNPox having a more substantial antiadhesive effect, while GO was, additionally, very effective at killing bacteria. This might be explained by the smaller surface area of GO sheets compared to GNPox, as observed in our previous studies,^57,58^ and their higher defect density, which causes more oxidative stress and, ultimately, bacterial death.

In sum, the antimicrobial action of our surface is based on antiadhesion properties, preventing bacterial attachment and subsequent biofilm formation, except in the case of GO-containing coatings, which also promote bacterial death. Inhibition hampers bacteria attachment to the surface, preventing the establishment of permanent interactions between the surface and the bacteria membrane. Clinically, inhibiting bacterial adhesion can significantly reduce the risk of Healthcare-Associated Infections (HAIs), lower antibiotic use, and improve patient outcomes, particularly in medical device applications.

### 3.5 Clotting Time Evaluation

Surface-induced blood clotting is one of the leading challenges related to blood-contacting biomedical devices and biomaterials. Clotting times of recalcified plasma were evaluated for the spray coatings with GNPox and GO, and the results are depicted in Figure 6. The recalcified plasma solutions in blank wells (TCPS) and in contact with PP did not coagulate completely by the end of the period tested (7 h). Oppositely, in contact with the glass disks, plasma solutions coagulated in 5 min, having the shortest clotting time and suggesting that glass was potent in activating the intrinsic coagulation cascade. This was expected, since it is well-known that surfaces such as glass initiate the intrinsic coagulation pathway via contact activation by adsorbing the responsible factors (factors XI and XII, high-molecular-weight kininogen HMWK, and prekallikrein, PK).^59^ Uncoated PU films had a clotting time of 19 min, and similar values of around 13 min were reported in other studies.^34^ Even though there is an increase of the clotting time of the uncoated compared to glass (*p* < 0.05), there are no statistically significant differences between uncoated and coated films. Since clotting times from spray-coated samples were similar to the control bare PU (uncoated), spray coating of GNPox and GO did not cause higher activation of protein adsorbed to the surfaces, consequent to their exposure to plasma, and did not increase the thrombogenicity under the tested conditions in PU. The surface modification by spray coating and the presence of the oxidized GBMs did not influence the thrombogenicity of PU and, therefore, PU spray-coated surfaces can be considered safe in terms of PU biomaterials-associated thrombosis.^6,59^ Although simple and effective, the clotting time assay used in this study has certain limitations that must be acknowledged. The most relevant include (i) the influence of the material where the experiment is being performed, i.e., the coated PU samples are placed inside a polystyrene multiwell plate that is always in contact with the plasma, and that, by itself, will induce coagulation (despite proper controls having been made to take this into account), and (ii) the lack of cellular interactions with immune cells and endothelial factors that release anticoagulant factors.

**Figure 6:**
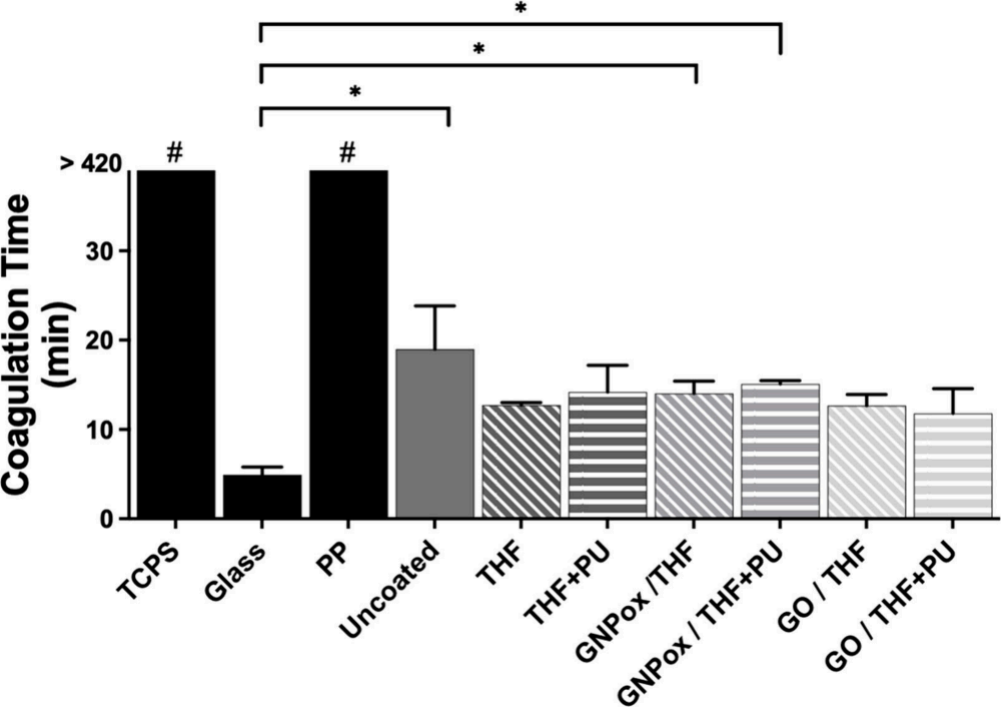
Clotting time of recalcified plasma in polystyrene (PS), glass, polypropylene (PP), uncoated PU, and spray coated surfaces. All samples (*n* = 3) were put in a 48-well non-tissue culture-treated PS plate (PS sample means the well plate only). Symbol ^#^ indicates that TCPS and PP are statistically significantly different from all other samples and * that glass is statistically significantly different from uncoated, GNPox/THF, and GNPox/THF+PU (Kruskal–Wallis; *p* ≤ 0.05).

GO’s oxidation introduces oxygen-containing functional groups, increasing its hydrophilicity and reducing its interaction with platelets and coagulation factors. This decreased surface reactivity may lower platelet adhesion and activation, potentially reducing the thrombotic risk. Several other studies have reported graphene as being hemocompatible.^60,61^ Hydrophobic graphene and hydrophilic functionalized graphene have shown excellent compatibility with red blood cells, platelets, and plasma coagulation pathways.^60^ Moreover, hydrophobic pristine graphene showed significantly reduced ROS-mediated toxicity effects on macrophages with the functionalization of graphene with hydrophilic groups.^60^ The use of GO also has little effect on the extrinsic coagulation pathway, and, when in small concentrations (0.001–0.05 mg/mL), however, slightly activating the clotting factors, it has little impact on the activity of fibrinogen polymerization and platelet aggregation.^62^ The hemocompatibility of GO is being explored to develop for example heparin-mimicking hydrogels^63^ and for antithrombotic applications.^61^ Therefore, GO and coatings containing GNPox or GO can be used for blood-contacting biomedical devices or materials.

### 3.6 *In Vitro* Biocompatibility

Biocompatibility of the spray coatings with GNPox and GO was evaluated *in vitro* by incubating cells with medium extracts of the samples. The possible cytotoxic effects of medium extracts were evaluated by measuring the metabolic activity of human fibroblasts (HFF-1 cells), and the results are depicted in Figure 7. Compared to the positive control, fibroblast metabolic activity was close to 100 (±6)% for all samples. For the negative control of cell death, cells cultured in DMEM+ with Triton 0.1%, metabolic activity was close to 0%. These results showed that none of the obtained extracts affected the cell metabolic activity, demonstrating the lack of cytotoxicity of the coatings and confirming the possibility of their use for biomedical applications. Toxicity can be caused by the leaching of the materials,^64^ and these results suggest that spraying promoted a well-adhered coating of GBMs at the surface of PU.

**Figure 7:**
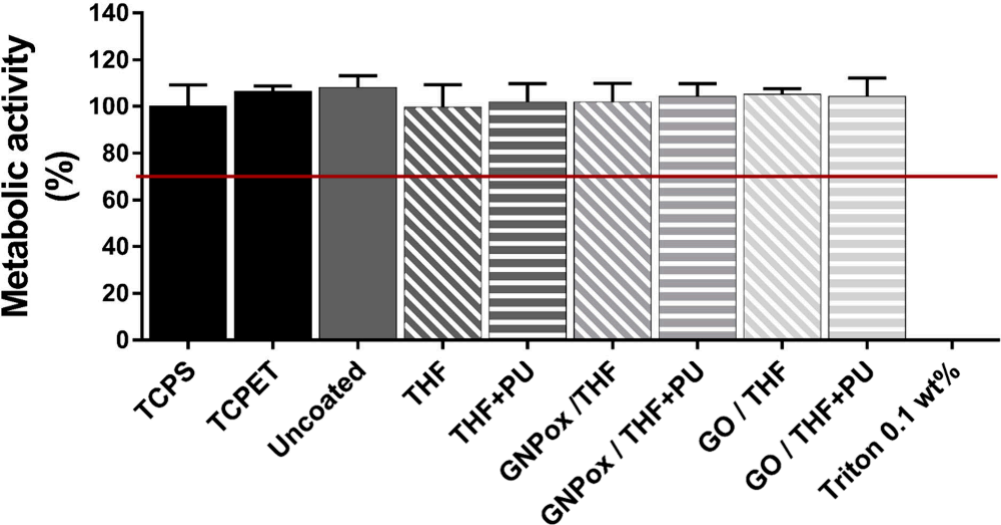
Metabolic activity of HFF-1 cells after incubation with medium extracts (using DMEM+ as an extraction vehicle) for 24 h. Cell metabolic activity is represented as a percentage compared to cells growing in DMEM+ (100%). DMEM+ subjected to the same conditions of the extracts was used as positive control, while a solution of DMEM+ with Triton X-100 0.1 wt % was used as a negative control. The red line at 70% marks the limit for considering the material extracts cytotoxic, according to ISO 10993-5. No statistically significant differences were found between samples, only for the negative control (Kruskal–Wallis; *p* ≤ 0.05).

Biocompatibility was further studied by culturing cells at the materials’ surfaces in a direct contact assay for 24 h, evaluating cell adhesion and morphology (Figure 8, Supporting Information Figure S5). In the positive control of cells cultured directly in the well (TCPS), a high cell density was observed, with fibroblasts in a spindle-like shape, the typical morphology of these cells. In PU surfaces, with or without spraying (uncoated, THF, THF+PU), cell adhesion was decreased with very few cells found adhered at the surface, in a round-shaped conformation, and less spread. On the other hand, in the surfaces with oxidized GBMs, the cell population density was much higher, mainly in aggregates, and cells were more elongated than in PU. Comparing coatings without versus with PU in the solution, the presence of PU seemed to decrease cell adhesion and cells had a more rounded morphology. This effect is likely due to the inherent hydrophobicity of PU, which may adsorb lower quantities of extracellular adhesion proteins (fibronectin and vitronectin, in particular) or cause them to undergo conformational changes, making them less bioactive. When adsorbed, such proteins mediate cell response to biomaterials such as cell adhesion, morphology, and migration.^65^ Higher surface hydrophilicity favors vitronectin adhesion and allows fibronectin to retain its functionality; additionally, an increase in surface roughness and topographic features may lead to an increase in fibronectin adsorption due to an increase in surface area.^66^ This effect is likely due to the inherent hydrophobicity of PU, which reduces protein adsorption, particularly extracellular adhesion proteins like fibronectin and vitronectin, which are essential for cell attachment and spreading. On PU-rich surfaces these proteins may adsorb in smaller amounts or undergo conformational changes, diminishing their bioactivity. In contrast, coatings sprayed with GNPox or GO alone (i.e., GNPox/THF and GO/THF) promoted higher cell density and the cells exhibited a more elongated morphology. This is likely attributed to the oxygen-containing functional groups present in these materials, which increase surface hydrophilicity, polarity, and protein adsorption.^66^ The improved wettability of GNPox and GO coatings may also facilitate better adsorption and retention of adhesion proteins, enhancing the cell–material interactions. Additionally, an increase in the surface roughness and topographic features on oxidized GBMs coatings may contribute to higher fibronectin adsorption, further promoting fibroblast attachment. These results suggest that while PU may offer structural benefits, optimizing the balance between PU content and oxidized graphene materials could enhance both antibacterial properties and biocompatibility. Pinto et al.^67^ also observed higher fibroblast adhesion and proliferation on poly­(lactic acid) (PLA)/GO films than in pristine PLA. Similarly, Ruiz et al.^68^ observed that human adenocarcinoma HT-29 cells attached and grew more in GO-coated glass slides, compared to the control (uncoated glass slides), and stated that GO acted as a scaffold for cell attachment and proliferation. In a different approach, Zhao and co-workers^65^ studied GO-based coatings on nitinol substrates and found that coatings containing GO combined antimicrobial activity toward *E. coli* with improved biocompatibility, showing higher cell adhesion, proliferation, and differentiation of mouse osteoblastic cells.

**Figure 8:**
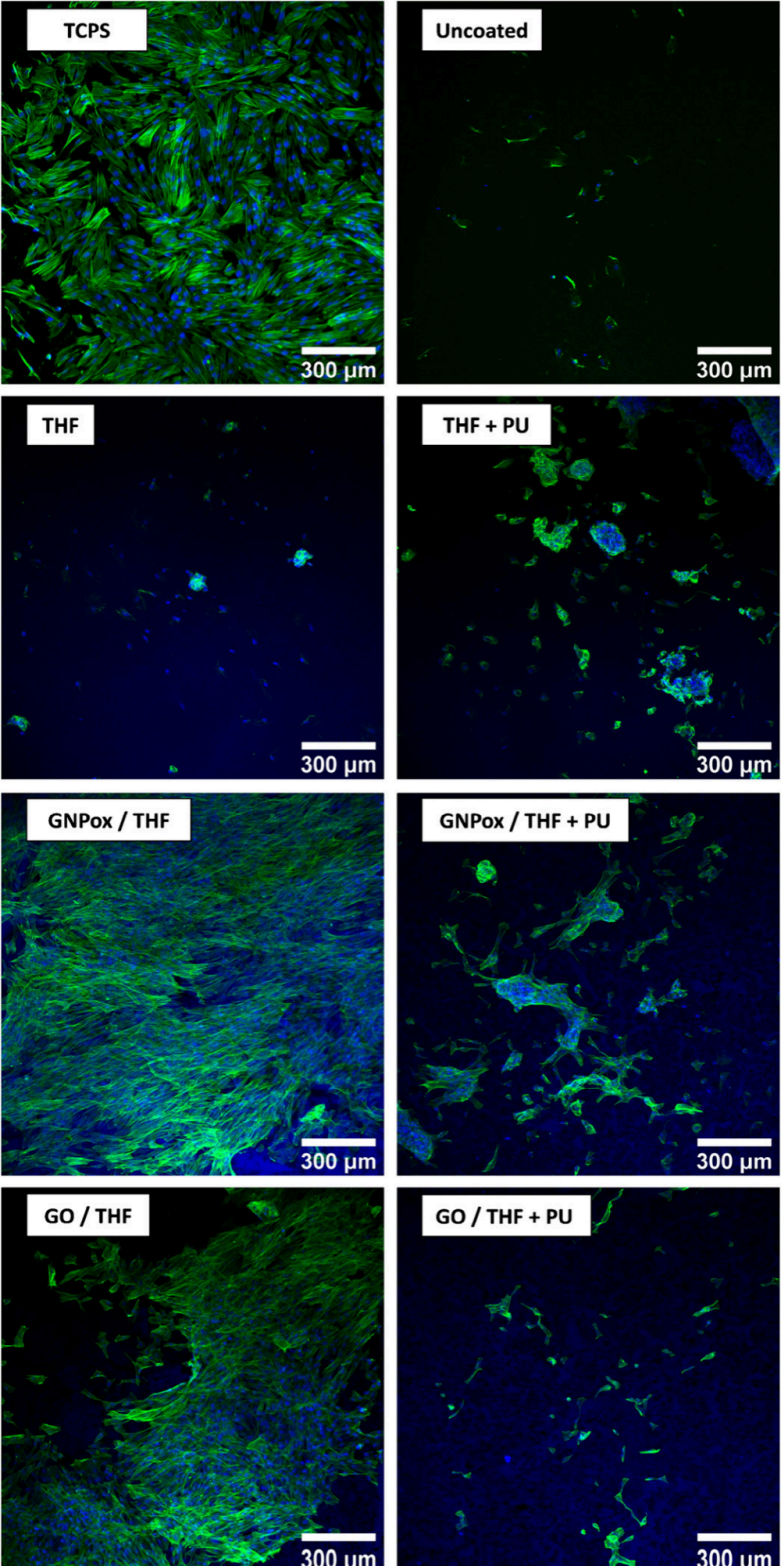
Representative immunofluorescence images of HFF-1 cells cultured in a spray coating surface for 24 h. Fibroblasts were stained with DAPI (nuclei, blue) and phalloidin (F-actin in the cytoskeleton, green). The scale bar represents 300 μm.

Fibroblasts, the most common mammalian connective tissue cells, are found in most tissues, including the dermis (dermal fibroblasts).^69^ Therefore, our results suggest that the contact of materials coated with the produced coatings containing GNPox and GO should not cause adverse effects upon contact with the skin, which may be the case for most of the applications.

As stated by Böswald et al.,^7^ infection-resistant materials must possess the following properties: 1) antimicrobial activity against a broad spectrum, which can be achieved by any combination of reduction or prevention of adherence of bacterial and fungal microorganisms; reduction or prevention of biofilm formation; bacteriostatic and/or bactericidal properties; 2) adequate, long-term duration of antimicrobial activity; 3) antimicrobial activity must be present on the outer and inner surfaces of the device; 4) the polymer modification must not affect the physical properties of the biomaterial, i.e., stability, durability, elasticity and strength, thrombogenicity, and cytotoxicity. Therefore, this study should be considered as a preliminary evaluation or exploratory study of the potential application of graphene-based materials through spray coating methods as antibacterial coatings.

As previously mentioned, certain surface characteristics of our GBMox coatings, like an average roughness (*S*_a_) below 200 nm and hydrophilicity below 90°, are particularly advantageous in biomedical applications, such as for blood-contacting devices, because they reduce bacterial adhesion. This can have profound implications for both patient outcomes and healthcare practices. One example of real-world applications is their use for medical implants and devices, where smooth, hydrophilic surfaces on prosthetics, catheters, and other devices can significantly lower the risk of bacterial infection. Infections associated with implants often require extensive treatments like antibiotics or removal of the device, increasing patient discomfort and healthcare costs. Particularly in the case of catheters, the decreased and selective protein adsorption on hydrophilic materials may also improve thromboresistance, reducing clot formation and enhancing blood compatibility, which is crucial for implants and devices in contact with blood.^70^ Another example is its application on hospital-acquired infections, since surfaces in hospital environments that reduce bacterial adhesion (e.g., surgical tools, countertops) help combat HAIs, which are a major cause of morbidity and mortality. This coating technology could therefore be applied to a wide range of applications where bacterial adhesion is an issue.

Although this study confirms the biocompatibility and antibacterial performance of GNPox and GO coatings, further research could complement our findings. Evaluating their long-term stability under physiological conditions would provide insights into their durability for clinical applications. Additional hemocompatibility assessments, such as hemolysis assays, platelet adhesion and activation tests, and dynamic flow studies, could further support their safety in blood-contacting environments. Moreover, testing their antimicrobial efficacy against a broader range of clinically relevant pathogens, including antibiotic-resistant strains, would reinforce their potential for infection-resistant medical applications. These investigations are important for the successful translation of GNPox and GO coatings into commercial medical devices that combine effective bacterial resistance with excellent blood compatibility.

## 4 Conclusions

The spray coating method is an easy, reproducible, and expedited procedure. It also yields homogeneous surfaces with materials evenly distributed along the coated surface. This marks a significant advantage over dip coating, which has a very low yield of randomly distributed individual particles. For these reasons, we believe that our spraying method has the potential for the large-scale coating of oxidized graphene on PU surfaces, such as in hemodialysis catheters.

The coatings containing GNPox and GO have been shown to adhere well to the PU substrate, which is critical to avoid any adverse effects and for a long-term duration of the antibacterial activity. The use of oxidized GBMs increased the hydrophilicity of the surfaces, which influenced on one hand the decrease of *S. epidermidis* and, on the other hand, the increase of fibroblast adhesion. By evaluating the antibacterial activity, coatings containing GNPox are more promising, significantly decreasing bacterial adhesion up to 82% (*p* < 0.05). The use of dissolved PU as binder in the coating solution has shown to be irrelevant in the wettability and antibacterial activity of the surfaces, as well as in terms of GBMs adhesion to the substrate. However, fibroblast adhesion seems to be favored in coatings that do not contain PU. The application of these coatings did not alter the thrombogenicity of the bare PU, and their biocompatibility was proved by evaluating coating medium extracts and by direct contact with fibroblasts (HFF-1). This work reinforces the use of oxidized graphene-based materials as a multifunctional platform to develop antimicrobial polymeric surfaces that prevent or resist bacterial colonization, while ensuring biocompatibility and cell spreading without causing adverse effects. The incorporation of GO and GNPox in polyurethane-based coatings represents a transformative advancement. Their mechanical durability, antibacterial effects, biocompatibility, and coagulation-free properties offer a superior alternative to conventional uncoated PU. Ultimately, these oxidized graphene-based coatings offer a versatile solution for infection control and biocompatibility in blood-contacting devices and potential applications extending beyond the healthcare sector, due to their potential to provide quality and safety benefits to many materials.

These findings warrant further studies and refinements to advance the clinical application of graphene-based antibacterial coatings.

## Supplementary Material





## Notes

Figure S1, SEM images of the uncoated and coated films’ transversal cuts; Figure S2, SEM images of the coated films’ surface before and after the rubbing test was applied; Figure S3, the antibacterial activity of polypropylene control film in comparison to TCPET- and GO/THF-coated films toward adherent *S. epidermidis* after 24 h of incubation in unsupplemented culture medium; Figure S4, metabolic activity of planktonic bacteria; Figure S5, representative immunofluorescence images of HFF-1 cells cultured in spray-coated surfaces for 24 h (PDF)
Supporting Information table (XLSX)

## ABBREVIATIONS

2D: two-dimensional
3D: three-dimensional
AFM: atomic force microscope
ANOVA: analysis of variance
AOBS: acousto-optical beam splitter
CEMUP: Centro de Materiais da Universidade do Porto
CFUs: colony forming units
4′,6-diamidino-2-phenylindole dihydrochloride: DAPI
*d*H_2_O: distilled water
DMEM: Dulbecco’s modified Eagle medium
DMF: dimethylformamide
DNA: deoxyribonucleic acid
EDTA: ethylenediaminetetraacetic acid
*Escherichia coli*: *E. coli*
ETD: Everhart-Thornley detector
FBS: fetal bovine serum
FLG, few-layer graphene GBMox: oxidized graphene-based materials
GBMs: graphene-based materials
GNPox: oxidized graphene nanoplatelets
GO: graphene oxide
HV: high voltage
mag: magnification
PBS: phosphate-buffered saline
PI: propidium iodide
PLA: poly­(lactic acid)
PP: polypropylene
PS: polystyrene
PU: polyurethane
RFUs: relative fluorescence units
RNA: ribonucleic acid
ROS: reactive oxygen species
rpm: revolutions per minute
RT: room temperature
SED: secondary electron detector
SEM: scanning electron microscopy
TCPET: tissue culture polyethylene terephthalate
THF: tetrahydrofuran
TSB: trypticase soy broth
XPS: X-ray photoelectron spectroscopy
WD: working distance

## References

[1] Altun, E. ; Aydogdu, M. O. ; Chung, E. ; Ren, G. ; Homer-Vanniasinkam, S. ; Edirisinghe, M. Metal-based nanoparticles for combating antibiotic resistance. Applied Physics Reviews 2021, 8, 10.1063/5.0060299.

[2] Kenawy E.-R., Worley S. D., Broughton R. (2007). The Chemistry
and Applications of
Antimicrobial Polymers: A State-of-the-Art Review. Biomacromolecules.

[3] Szycher, M. High Performance Biomaterials: A Complete Guide to Medical and Pharmceutical Applications; CRC Press: 1991.

[4] Szycher, M. Polyurethanes: Antimicrobial. In Encyclopedia of Biomedical Polymers and Polymeric Biomaterials, 11 Vol. Set; CRC Press: 2015; pp 6610–6629.

[5] Wang, W. ; Wang, C. 3 - Polyurethane for biomedical applications: A review of recent developments. In The Design and Manufacture of Medical Devices; Davim, J. P. , Ed.; Woodhead Publishing: 2012; pp 115–151.

[6] Lelah M. D., Grasel T. G., Pierce J. A., Cooper S. L. (1986). Ex vivo interactions
and surface property relationships of polyetherurethanes. J. Biomed. Mater. Res..

[7] Böswald M., Girisch M., Greil J., Spies T., Stehr K., Krall T., Guggenbichler J.-P. (1995). Antimicrobial activity and biocompatibility
of polyurethane and silicone catheters containing low concentrations
of silver: A new perspective in prevention of polymer-associated foreign-body-infections. Zentralbl. Bakteriol..

[8] Burroughs L., Ashraf W., Singh S., Martinez-Pomares L., Bayston R., Hook A. L. (2020). Development of dual anti-biofilm
and anti-bacterial medical devices. Biomater.
Sci..

[9] Bander, S. J. ; Schwab, S. J. ; Woo, K. Central catheters for acute and chronic hemodialysis access. August 29th, 2016 ed.; Cull, D. L. ; Berns, J. S. , UpToDate library, 2016.

[10] Ibeas-Lopez J. (2015). New technology:
heparin and antimicrobial-coated catheters. J. Vasc Access.

[11] Turnbull, I. R. ; Buckman, S. A. ; Horn, C. B. ; Bochicchio, G. V. ; Mazuski, J. E. Antibiotic-Impregnated Central Venous Catheters Do Not Change Antibiotic Resistance Patterns. Surg. Infect. 2018, 19, 40 10.1089/sur.2017.087. 29028461

[12] Rose S., Okere S., Hanlon G., Lloyd A., Lewis A. (2005). Bacterial
adhesion to phosphorylcholine-based polymers with varying cationic
charge and the effect of heparin pre-adsorption. J. Mater. Sci.: Mater. Med..

[13] Jennings J. A., Carpenter D. P., Troxel K. S., Beenken K. E., Smeltzer M. S., Courtney H. S., Haggard W. O. (2015). Novel antibiotic-loaded
point-of-care
implant coating inhibits biofilm. Clinical Orthopaedics
and Related Research.

[14] Szycher, M. Antimicrobial Materials for Medical Devices. Kirk-Othmer Encyclopedia of Chemical Technology 2015, 1 10.1002/0471238961.koe00009.

[15] Liu Z., Yi Y., Song L., Chen Y., Tian L., Zhao J., Ren L. (2022). Biocompatible
mechano-bactericidal nanopatterned surfaces with salt-responsive
bacterial release. Acta Biomaterialia.

[16] Miao J., Chai H., Niu L., Ouyang M., Wang R. (2025). A stable dual
functional superhydrophobic coating to inhibit Proteus mirabilis colonization,
migration, and encrustation formation for urinary catheter applications. J. Mater. Chem. B.

[17] Wei T., Tang Z., Yu Q., Chen H. (2017). Smart antibacterial
surfaces with switchable bacteria-killing and bacteria-releasing capabilities. ACS Appl. Mater. Interfaces.

[18] Pan X., Sun Y., Lin Y., Chen X., Kong X., Gao Q., Xu Y., Zhang L. (2024). An Intelligent Antibacterial Hydrogel Coating with
Triple Antifouling–Killing–Releasing Functions for Medical
Tubing. ACS Applied Polymer Materials.

[19] Wang L., Guo X., Zhang H., Liu Y., Wang Y., Liu K., Liang H., Ming W. (2022). Recent advances
in superhydrophobic
and antibacterial coatings for biomedical materials. Coatings.

[20] Li Z., Zhang W., Luo Y., Yang J., Hou J. G. (2009). How Graphene
Is Cut upon Oxidation?. J. Am. Chem. Soc..

[21] Henriques P. C., Borges I., Pinto A. M., Magalhães F. D., Gonçalves I. C. (2018). Fabrication and antimicrobial performance
of surfaces
integrating graphene-based materials. Carbon.

[22] Gomes R. N., Borges I., Pereira A. T., Maia A. F., Pestana M., Magalhães F. D., Pinto A. M., Gonçalves I. C. (2018). Antimicrobial
graphene nanoplatelets coatings for silicone catheters. Carbon.

[23] Yousefi M., Dadashpour M., Hejazi M., Hasanzadeh M., Behnam B., de la Guardia M., Shadjou N., Mokhtarzadeh A. (2017). Anti-bacterial
activity of graphene oxide as a new weapon nanomaterial to combat
multidrug-resistance bacteria. Mater. Sci. Eng.
C Mater. Biol. Appl..

[24] Henriques P. C., Pereira A. T., Bogas D., Fernandes J. R., Pinto A. M., Magalhães F. D., Gonçalves I. C. (2021). Graphene
films irradiated with safe low-power NIR-emitting diodes kill multidrug
resistant bacteria. Carbon.

[25] Hegab H. M., ElMekawy A., Zou L., Mulcahy D., Saint C. P., Ginic-Markovic M. (2016). The controversial
antibacterial activity of graphene-based
materials. Carbon.

[26] Perreault F., de Faria A. F., Nejati S., Elimelech M. (2015). Antimicrobial
properties of graphene oxide nanosheets: why size matters. ACS Nano.

[27] Borges I., Henriques P. C., Gomes R. N., Pinto A. M., Pestana M., Magalhães F. D., Gonçalves I. C. (2020). Exposure of smaller and oxidized
graphene on polyurethane surface improves its antimicrobial performance. Nanomaterials.

[28] Schlaich C., Li M., Cheng C., Donskyi I. S., Yu L., Song G., Osorio E., Wei Q., Haag R. (2018). Mussel-inspired polymer-based
universal spray coating for surface modification: fast fabrication
of antibacterial and superhydrophobic surface coatings. Adv. Mater. Interfaces.

[29] Hu W., Peng C., Luo W., Lv M., Li X., Li D., Huang Q., Fan C. (2010). Graphene-Based Antibacterial Paper. ACS Nano.

[30] Ganguly A., Sharma S., Papakonstantinou P., Hamilton J. (2011). Probing the thermal
deoxygenation of graphene oxide using high-resolution in situ X-ray-based
spectroscopies. J. Phys. Chem. C.

[31] Kovtun A., Jones D., Dell’Elce S., Treossi E., Liscio A., Palermo V. (2019). Accurate chemical analysis
of oxygenated graphene-based
materials using X-ray photoelectron spectroscopy. Carbon.

[32] Schienle S., Al-Ahmad A., Kohal R. J., Bernsmann F., Adolfsson E., Montanaro L., Palmero P., Fürderer T., Chevalier J., Hellwig E., Karygianni L. (2016). Microbial
adhesion on novel yttria-stabilized tetragonal zirconia (Y-TZP) implant
surfaces with nitrogen-doped hydrogenated amorphous carbon (a-C:H:N)
coatings. Clin. Oral Investig..

[33] Crawford R. J., Webb H. K., Truong V. K., Hasan J., Ivanova E. P. (2012). Surface
topographical factors influencing bacterial attachment. Adv. Colloid Interface Sci..

[34] Goncalves I. C., Martins M. C., Barbosa M. A., Ratner B. D. (2009). Protein adsorption
and clotting time of pHEMA hydrogels modified with C18 ligands to
adsorb albumin selectively and reversibly. Biomaterials.

[35] Liu Y. M., Wen J., Gao Y., Li T. Y., Wang H. F., Yan H., Niu B. L., Guo R. J. (2018). Antibacterial graphene oxide coatings
on polymer substrate. Appl. Surf. Sci..

[36] Thampi S., Nandkumar A. M., Muthuvijayan V., Parameswaran R. (2017). Differential
adhesive and bioactive properties of the polymeric surface coated
with graphene oxide thin film. ACS Appl. Mater.
Interfaces.

[37] Bandeira P., Monteiro J., Baptista A. M., Magalhães F. D. (2016). Influence
of oxidized graphene nanoplatelets and [DMIM]­[NTf_2_] ionic
liquid on the tribological performance of an epoxy-PTFE coating. Tribiol. Int..

[38] Tang H., Cao T., Liang X., Wang A., Salley S. O., McAllister J., Ng K. Y. S. (2009). Influence of silicone surface roughness and hydrophobicity
on adhesion and colonization ofStaphylococcus epidermidis. J. Biomed. Mater. Res. Part A.

[39] Butler J., Handy R. D., Upton M., Besinis A. (2023). Review of antimicrobial
nanocoatings in medicine and dentistry: mechanisms of action, biocompatibility
performance, safety, and benefits compared to antibiotics. ACS Nano.

[40] Nguyen D. H., Pham V. T., Truong V. K., Sbarski I., Wang J., Balčytis A., Juodkazis S., Mainwaring D. E., Crawford R. J., Ivanova E. P. (2018). Role of topological scale in the
differential fouling of Pseudomonas aeruginosa and Staphylococcus
aureus bacterial cells on wrinkled gold-coated polystyrene surfaces. Nanoscale.

[41] Šístková J., Fialová T., Svoboda E., Varmužová K., Uher M., Číhalová K., Přibyl J., Dlouhý A., Pávková
Goldbergová M. (2024). Insight into antibacterial effect of titanium nanotubular
surfaces with focus on Staphylococcus aureus and Pseudomonas aeruginosa. Sci. Rep..

[42] De-la-Pinta I., Cobos M., Ibarretxe J., Montoya E., Eraso E., Guraya T., Quindós G. (2019). Effect of
biomaterials hydrophobicity
and roughness on biofilm development. J. Mater.
Sci.: Mater. Med..

[43] Rochford E., Poulsson A., Varela J. S., Lezuo P., Richards R., Moriarty T. (2014). Bacterial adhesion to orthopaedic
implant materials
and a novel oxygen plasma modified PEEK surface. Colloids Surf. B Biointerfaces.

[44] Webb H. K., Boshkovikj C. J., Fluke C. J., Truong V. K., Hasan J., Baulin V. A., Lapovok R., Estrin Y., Crawford R. J., Ivanova E. P. (2013). Bacterial
attachment on sub-nanometrically smooth titanium
substrata. Biofouling.

[45] Raulio M., Järn M., Ahola J., Peltonen J., Rosenholm J. B., Tervakangas S., Kolehmainen J., Ruokolainen T., Narko P., Salkinoja-Salonen M. (2008). Microbe repelling coated stainless
steel analysed by field emission scanning electron microscopy and
physicochemical methods. JIMB.

[46] Peltonen J., Järn M., Areva S., Linden M., Rosenholm J. B. (2004). Topographical
Parameters for Specifying a Three-Dimensional Surface. Langmuir.

[47] Braem A., Mellaert L. V., Mattheys T., Hofmans D., Waelheyns E. D., Geris L., Anné J., Schrooten J., Vleugels J. (2014). Staphylococcal biofilm growth on smooth and porous
titanium coatings for biomedical applications. Jouranl of Biomedical Materials Research: Part A.

[48] Ghumatkar A., Budhe S., Sekhar R., Banea M., Barros S. d. (2016). Influence
of adherend surface roughness on the adhesive bond strength. Latin American Journal of Solids and Structures.

[49] Aissou T., Jann J., Faucheux N., Fortier L.-C., Braidy N., Veilleux J. (2023). Suspension plasma sprayed
copper-graphene coatings
for improved antibacterial properties. Appl.
Surf. Sci..

[50] Henriques P. c. C., Pereira A. T., Pires A. L., Pereira A. M., Magalhães F. D., Gonçalves I. s. C. (2020). Graphene surfaces interaction with
proteins, bacteria, mammalian cells, and blood constituents: The impact
of graphene platelet oxidation and thickness. ACS Appl. Mater. Interfaces.

[51] Michels R., Last K., Becker S. L., Papan C. (2021). Update on coagulase-negative
staphylococci—what the clinician should know. Microorganisms.

[52] Feng G., Cheng Y., Wang S.-Y., Borca-Tasciuc D. A., Worobo R. W., Moraru C. I. (2015). Bacterial attachment and biofilm
formation on surfaces are reduced by small-diameter nanoscale pores:
how small is small enough?. Npj Biofilms And
Microbiomes.

[53] Hsu L. C., Fang J., Borca-Tasciuc D. A., Worobo R. W., Moraru C. I. (2013). Effect
of Micro- and Nanoscale Topography on the Adhesion of Bacterial Cells
to Solid Surfaces. Appl. Environ. Microbiol..

[54] Zou F., Zhou H., Jeong D. Y., Kwon J., Eom S. U., Park T. J., Hong S. W., Lee J. (2017). Wrinkled surface-mediated
antibacterial activity of graphene oxide nanosheets. ACS Appl. Mater. Interfaces.

[55] Parra, C. A.-O. ; Montero-Silva, F. A.-O. ; Gentil, D. ; Del Campo, V. ; Henrique Rodrigues da Cunha, T. ; Henriquez, R. ; Haberle, P. A.-O. X. ; Garin, C. ; Ramirez, C. ; Fuentes, R. A.-O. ; Flores, M. ; Seeger, M. The Many Faces of Graphene as Protection Barrier. Performance under Microbial Corrosion and Ni Allergy Conditions. Materials 2017, 10, 1406 10.3390/ma10121406. 29292763 PMC5744341

[56] Shih S.-J., Hong B.-J., Lin Y.-C. (2017). Novel graphene
oxide-containing antibacterial
mesoporous bioactive glass. Ceram. Int..

[57] Pinto A. M., Cabral J., Tanaka D. a. P., Mendes A. M. A. M., Magalhães F. D., Magalhaes F. D. (2013). Effect of incorporation of graphene
oxide and graphene nanoplatelets on mechanical and gas permeability
properties of poly­(lactic acid) films. Polym.
Int..

[58] Pinto A. M., Gonçalves C., Sousa D. M., Ferreira A. R., Moreira J. A., Gonçalves I. C., Magalhães F. D. (2016). Smaller particle size and higher
oxidation improves biocompatibility of graphene-based materials. Carbon.

[59] van
der Kamp K. W. H. J., Hauch K. D., Feijen J., Horbett T. A. (1995). Contact
activation during incubation of five different polyurethanes or glass
in plasma. J. Biomed. Mater. Res..

[60] Sasidharan A., Panchakarla L. S., Sadanandan A. R., Ashokan A., Chandran P., Girish C. M., Menon D., Nair S. V., Rao C. N. R., Koyakutty M. (2012). Hemocompatibility
and Macrophage Response of Pristine
and Functionalized Graphene. Small.

[61] Kenry, Loh K. P., Lim C. T. (2015). Molecular
Hemocompatibility of Graphene Oxide and Its Implication for Antithrombotic
Applications. Small.

[62] Feng R., Yu Y., Shen C., Jiao Y., Zhou C. (2015). Impact of graphene
oxide on the structure and function of important multiple blood components
by a dose-dependent pattern. J. Biomed. Mater.
Res., Part A.

[63] He C., Shi Z.-Q., Ma L., Cheng C., Nie C.-X., Zhou M., Zhao C.-S. (2015). Graphene
oxide based heparin-mimicking
and hemocompatible polymeric hydrogels for versatile biomedical applications. J. Mater. Chem. B.

[64] Pinto A. M., Goncalves I. C., Magalhaes F. D. (2013). Graphene-based materials biocompatibility:
A review. Colloids Surf. B Biointerfaces.

[65] Zhao C., Pandit S., Fu Y., Mijakovic I., Jesorka A., Liu J. (2016). Graphene oxide based
coatings on
nitinol for biomedical implant applications: effectively promote mammalian
cell growth but kill bacteria. RSC Adv..

[66] Bacakova L., Filova E., Parizek M., Ruml T., Svorcik V. (2011). Modulation
of cell adhesion, proliferation and differentiation on materials designed
for body implants. Biotechnology Advances.

[67] Pinto A. M., Moreira S., Gonçalves I. C., Gama F. M., Mendes A. M., Magalhães F. D. (2013). Biocompatibility
of poly­(lactic acid) with incorporated
graphene-based materials. Colloids Surf. B Biointerfaces.

[68] Ruiz O. N., Fernando K. A., Wang B., Brown N. A., Luo P. G., McNamara N. D., Vangsness M., Sun Y. P., Bunker C. E. (2011). Graphene
oxide: a nonspecific enhancer of cellular growth. ACS Nano.

[69] Alberts, B. ; Johnson, A. ; Lewis, J. ; Raff, M. ; Roberts, K. ; Walter, P. Fibroblasts and Their Transformations: The Connective-Tissue Cell Family. In Molecular Biology of the Cell, 4th ed.; Garland Science, 2002.

[70] Kuo Z. K., Fang M. Y., Wu T. Y., Yang T., Tseng H. W., Chen C. C., Cheng C. M. (2018). Hydrophilic films: How hydrophilicity
affects blood compatibility and cellular compatibility. Advances in Polymer Technology.

