# Supplementary material for: Spray Coating Oxidized Graphene-Based Materials Makes Polyurethane Surfaces Antibacterial and Biocompatible: A Promising Approach for Medical Devices
Source: ACS Appl Mater Interfaces. 2026 Jan 20;18(3):4701–16. doi: 10.1021/acsami.4c20015 (PMC12862757; doi:10.1021/acsami.4c20015)
Supplement: Supplementary file 1 [file am4c20015_si_001.pdf]

Supporting Information

Spray coating oxidized graphene-based materials makes polyurethane surfaces antibacterial and biocompatible: a promising approach for medical devices

Natacha Rosa‡[a], Inês Borges‡[b,c], Patrícia C. Henriques[a,b,c], Andreia T. Pereira[b,c], Rita N. Gomes[b,c], Fernão D. Magalhães‡ [a,d] and Inês C. Gonçalves‡ [b,c]*

[a] LEPABE – Faculdade de Engenharia, Universidade do Porto, Rua Dr. Roberto Frias, 4200-465 Porto, Portugal

[b] i3S – Instituto de Investigação e Inovação em Saúde, Universidade do Porto, Rua Alfredo Allen, 208, 4200-135 Porto, Portugal

[c] INEB – Instituto de Engenharia Biomédica, Universidade do Porto, Rua Alfredo Allen, 208, 4200-135 Porto, Portugal

[d] ALiCE - Associate Laboratory in Chemical Engineering, Faculty of Engineering, University of Porto, Rua Dr. Roberto Frias, 4200-465 Porto, Portugal

Corresponding author: Inês C. Gonçalves icastro@i3s.up.pt

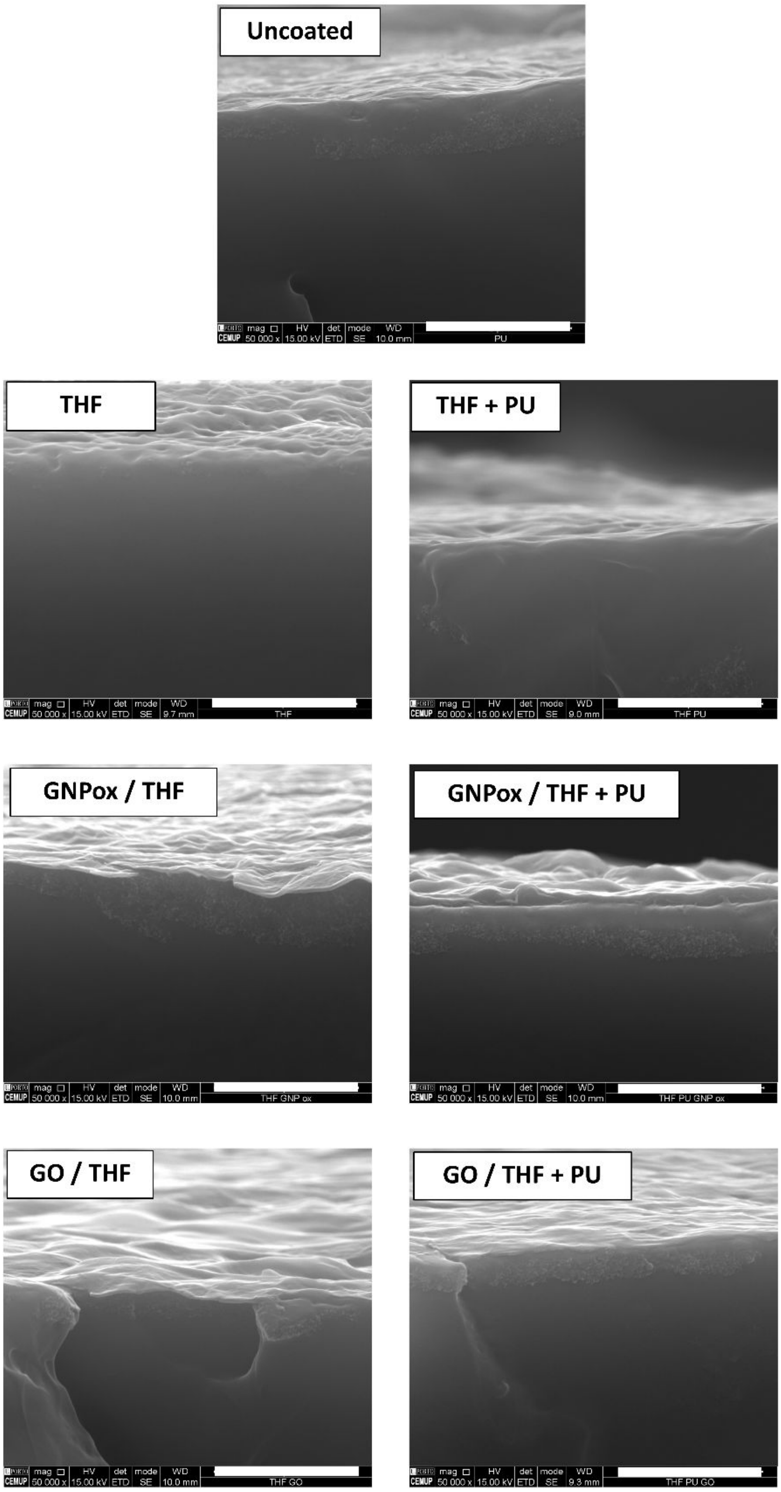

Figure S1. SEM images of the transversal cuts of uncoated and THF, THF+PU, GNPox/THF, GNPox/THF+PU, GO/THF and GO/THF+PU coated films. Images were

acquired using secondary electron detector (SE) imaging mode, voltage 15.00 kV and 50 000x magnification and working distance (WD) of approx.10 mm. Scale bar = 2 μm.

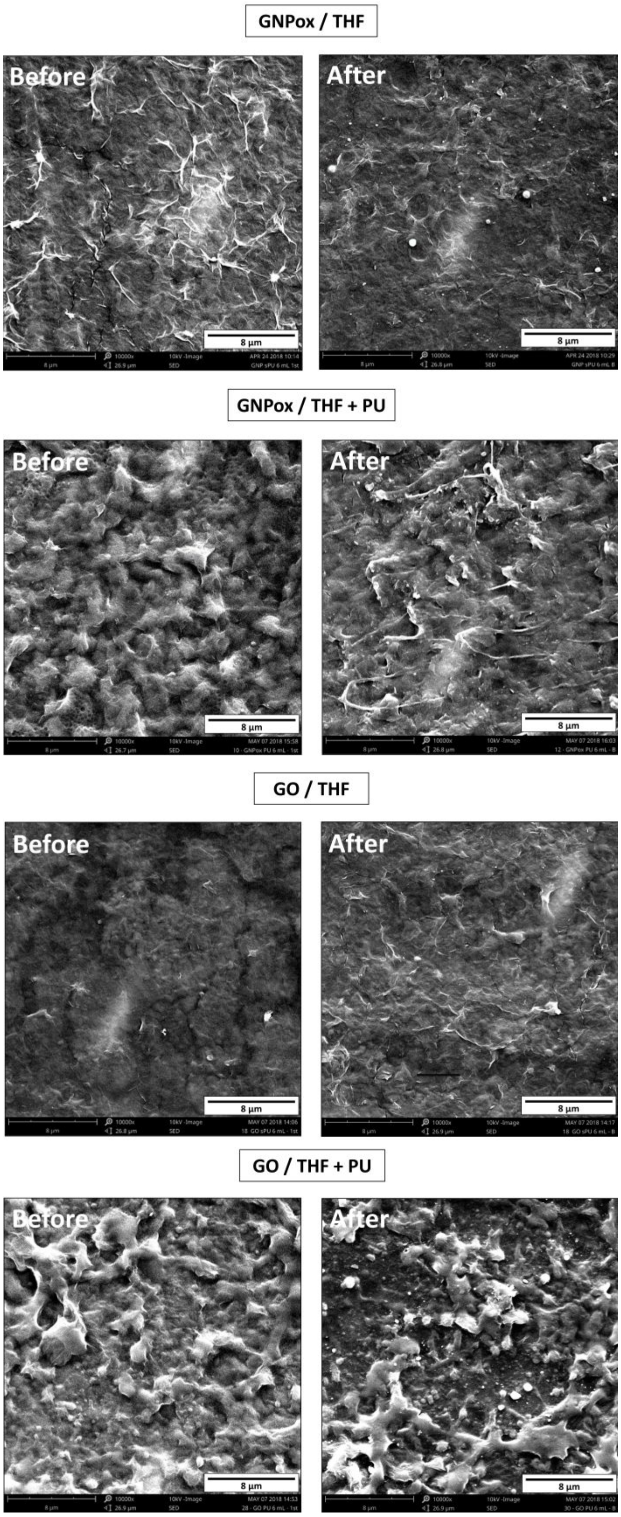

Figure S2. SEM images of the coated films surface before (on the left) and after (on the right) the rubbing test was applied. Images were acquired using secondary electron detector (SED), voltage 10.00 kV and 10 000x magnification. Scale bar = 8 µm.

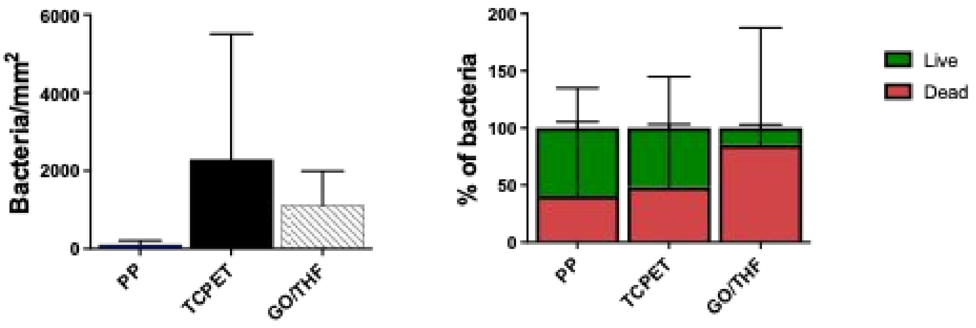

Figure S3 - Antibacterial activity of polypropylene (PP) control film in comparison to TCPET and GO/THF coated films towards adherent *S. epidermidis* after 24 h incubation in unsupplemented culture medium.

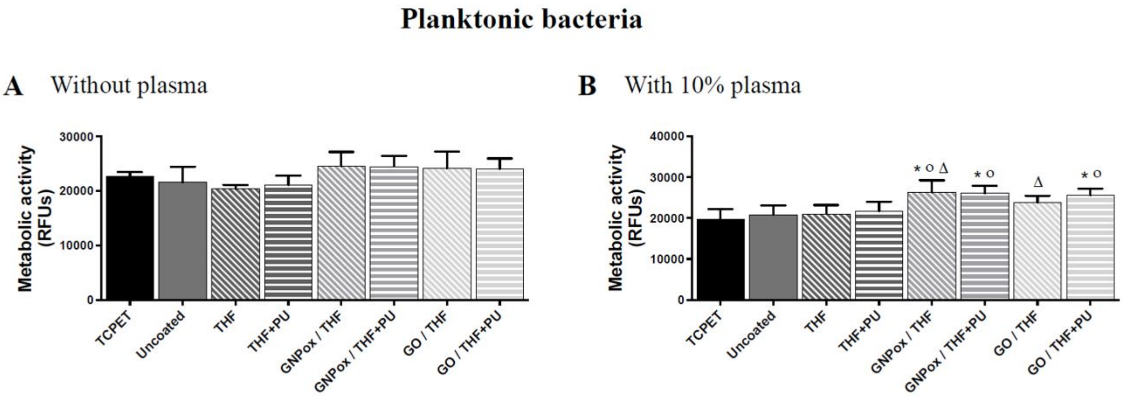

Figure S4 - Metabolic activity measured by Alamar Blue in planktonic bacteria (A) without plasma and supplemented (B) with 10% human plasma. The symbols indicate statistically significant differences compared to specific groups ($p \leq 0.05$; Kruskal

Wallis): * from TCPET, o from control PU film (Uncoated) and Δ from coating with THF and THF+PU.

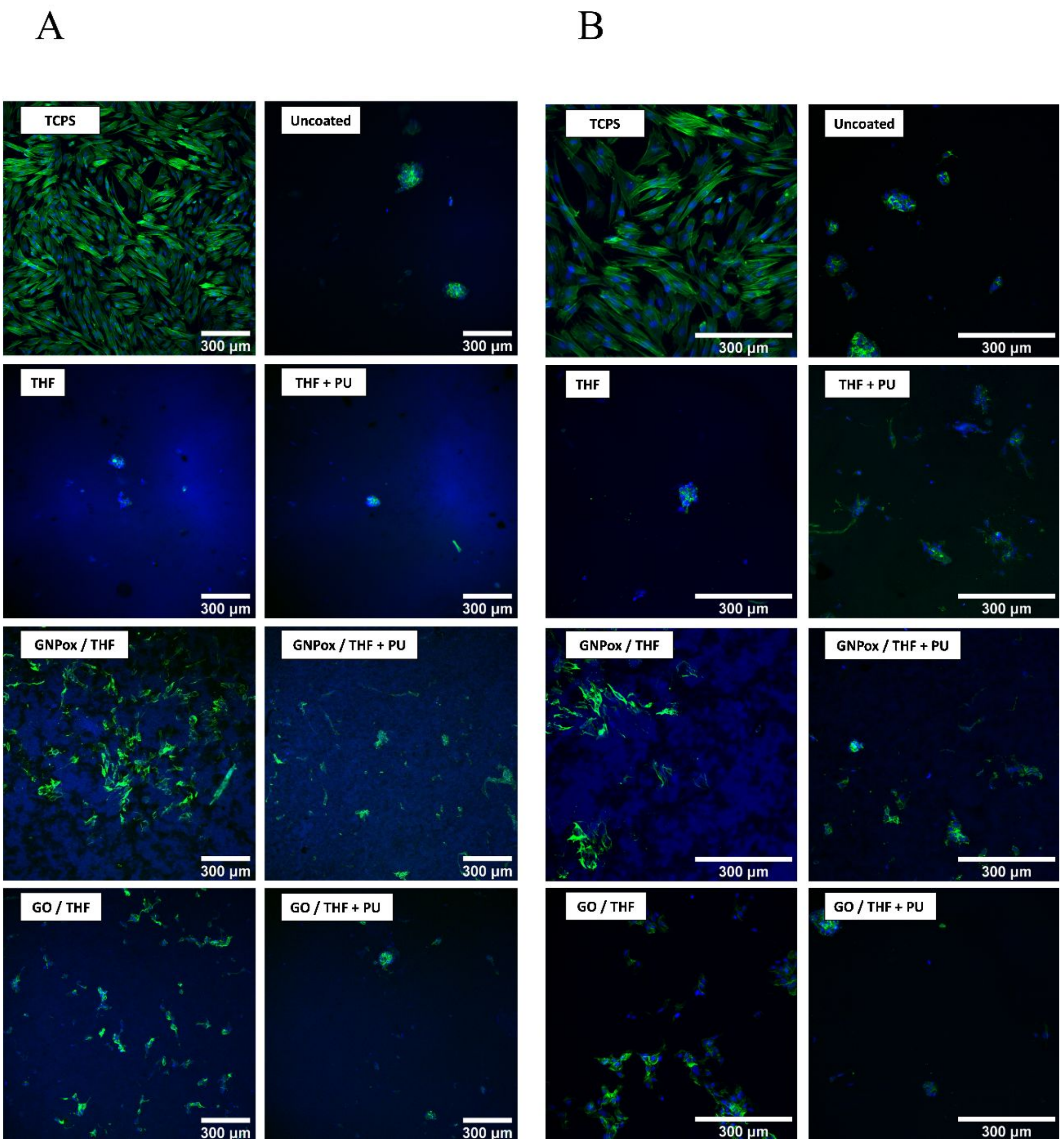

Figure S5. Representative immunofluorescence images of HFF-1 cells cultured in spray coating surfaces for 24 h. Here the direct contact assay was performed without pre-

seeding and 100 μL of a 1 ×105 cells/mL cell suspension were added per sample and incubated for 24 h. Fibroblasts were stained with DAPI (nuclei, blue) and phalloidin (F-actin in cytoskeleton, green). Magnification 10× (A) and 20× (B). Scale bar represents 300 μm.
